# GALNTL5, which is restricted to mouse spermatids, impairs endoplasmic reticulum (ER) function through direct interaction with ER chaperone proteins

**DOI:** 10.1038/s41420-024-02252-4

**Published:** 2024-12-18

**Authors:** Nobuyoshi Takasaki, Yoshihiro Koya, Mamoru Yamashita, Akihiro Nawa

**Affiliations:** 1 Department of Obstetrics and Gynecology Collaborative Research, Bell Research Center, Nagoya University Graduate School of Medicine, Nagoya, Japan; 2 Bell Research Center for Reproductive Health and Cancer, Kishokai Medical Corporation, Aichi, Japan

**Keywords:** Spermatogenesis, Cell death

## Abstract

Polypeptide *N*-acetylgalactosaminyltransferase-like protein 5 (*GALNTL5*) was identified as a pp-GalNAc-T family gene. Nevertheless, GALNTL5 has no glycosyltransferase activity. In mice, *Galntl5* expression is restricted to differentiating spermatids, and haploinsufficiency leads to immotile spermatozoa with an aberrant protein composition. Moreover, heterozygotic deletions of human *GALNTL5* have been detected in patients diagnosed with asthenozoospermia (low sperm motility). Although these findings indicate that GALNTL5 is a functional molecule essential for mature sperm formation in mammals, the exact function of GALNTL5 in spermiogenesis remains unknown. To clarify this role, we established the mouse spermatocyte cell line GC-2spd(ts), which exhibits drug-inducible GALNTL5 expression. Interestingly, continuous GALNTL5 expression in the resultant cell lines caused apoptosis with cell shrinkage, and GALNTL5 was localized in the endoplasmic reticulum (ER) and was associated with two ER-resident chaperone proteins, calnexin and BiP (GRP78). Calnexin recognized and strongly bound to the *N*-glycans on GALNTL5 molecules modified in the ER. In contrast, ER-resident BiP likely attached to GALNL5 regardless of its glycosylation. GALNTL5 expression abolished the binding between calnexin and misfolded substrate proteins, indicating that GALNTL5 directly blocks calnexin function. Furthermore, the interaction between GALNTL5 and calnexin decreased the level of BiP protein, and consequently also the expression levels of proteins that are resident in the ER, Golgi apparatus, and cytoplasm. These reduced protein levels were confirmed by loss of calnexin or BiP function in the GC-2spd(ts) cell line using siRNA knockdown. Further, sustained expression of GALNTL5 resulted in cell structure changes, including the position of the *cis*-Golgi apparatus and alterations in the ER network. These results strongly suggest that GALNTL5 contributes to alteration of the cell structure specific to differentiating spermatids by blocking ER function.

## Introduction

Spermatogenesis is a complex process in which spermatogonial stem cells form spermatozoa through the proliferative phase (spermatogonia), the meiotic phase (spermatocytes), and the differentiation or spermiogenic phase (spermatids). Analyses of model mice have shown that a large number of different genes contribute to the formation of fertilizable mature spermatozoa [1]. In the final spermiogenic stage after meiosis, round haploid spermatids differentiate into mature spermatozoa through dramatic morphological cells structure changes such as nuclear condensation, ER regression, flagellum formation, acrosome formation, and cytoplasmic renovation. These morphogenic transformation processes are well studied at the level of electron microscopy [2]. Regarding the formation of the acrosome, recent studies using knockout model mice have made it clear that many molecules comprising the Golgi apparatus contribute to acrosome biogenesis during spermiogenesis [3]. However, the molecular mechanisms of cell structure changes in spermiogenesis including the trigger for differentiation from round to elongated spermatids, remain unclear.

Thus far, mammalian glycosyltransferase genes have been comprehensively identified using a number of approaches, and the activities of various enzymes have been confirmed in vitro using biochemical methods [4]. One approach identified novel isoforms of the human *GALNTL5* and ortholog mouse *Galntl5* genes. Because GALNTL5 possesses highly conserved catalytic domains involved in transferring GalNAc from the nucleotide sugar to the acceptor residues, these genes are included in the polypeptide N-acetylgalactosaminytransferase (pp-GalNAc-T; EC 2.4.1.41) gene family [5]. However, GALNTL5 did not exhibit glycosyltransferase activity [6], probably because it uniquely lacks the C-terminal lectin domain conserved in this gene family. Interestingly, the expression of both human *GALNTL5* and mouse *Galntl5* genes is restricted to the testis. In situ hybridization confirmed that *GALNTL5* mRNA is expressed mainly in the round and elongated spermatids during spermiogenesis after meiosis, not in the outermost cells of the seminiferous tubules, which contain spermatogonia, spermatocytes, and somatic Sertoli cells [6]. More specifically, mouse GALNTL5 proteins were only detected in the juxta-nuclear space, and not in the acrosome, of round and elongating spermatids during spermiogenesis. Furthermore, mutant mice heterozygous for *Galntl5* were infertile, with impaired sperm motility and a high rate of morphological abnormalities resembling those seen in human asthenozoospermia. Mature sperm from mutant mice heterozygous for *Galntl5* exhibit decreased amounts of glycolytic enzymes required for motility, disruption of protein loading to acrosomes, and aberrant localization of the ubiquitin-proteasome system. By comparing the protein compositions of infertile human sperm on the basis of abnormalities in sperm from *Galntl5* heterozygous mutant mice, two asthenozoospermic patients were identified, each carrying one heterozygous nucleotide deletion in the human *GALNTL5* gene [6, 7]. These losses of function suggest that GALNTL5 is indispensable for mammalian spermiogenesis. However, its precise molecular role in mature sperm formation has remained unclear. To determine the function of GALNTL5 in mammalian spermiogenesis, we used cell lines derived from various tissues of different species with the goal of establishing cultured cells that constantly express GALNTL5. However, we have been unable to generate stable cell lines transfected with the mouse *Galntl5* gene. This strongly suggests that the GALNTL5 protein is toxic to all cell lines, affecting only differentiating spermatids that express it. In other words, the GALNTL5 protein functions only in mature sperm formation and should not be expressed in other cells or tissues.

It was recently reported that a human gene promotes apoptosis when overexpressed, actually inhibits the proliferation of cultured cells when gently expressed by drug induction [8]. Therefore, we sought to elucidate the role of GALNTL5 in spermiogenesis by establishing a cell line that gently expresses GALNTL5 upon chemical induction. In this study, we selected GC-2spd(ts) cells [9] because they are derived from mouse diploid spermatocytes that do not express *Galtntl5* mRNA and that have not yet differentiated into haploid spermatozoa expressing *Galtntl5* mRNA. Consequently, we were able to demonstrate that GALNTL5 induced apoptosis and the shrinkage of cultivated cells, that it was retained in the ER through its transmembrane domain, and that it impaired ER function via direct interactions with calnexin and BiP, both of which are chaperone proteins involved in ER homeostasis. Moreover, continuous GALNTL5 expression altered the structure of organelles, including the ER tubular structures and Golgi apparatus. Our data strongly indicate that GALNTL5 blocks ER function via its interaction with ER-resident chaperone proteins, and also suggest that it contributes to the differentiation of spermatids as a trigger of ER regression. Our successful establishment of drug-induced GALNTL5 expression in GC-2spd(ts) cells would lead to new hypotheses regarding the role of GALNTL5 in in vivo spermiogenesis.

## Results

### Establishment of the GC-2spd(ts) cell line expressing GALNTL5 upon chemical induction

**Fig. 1:**
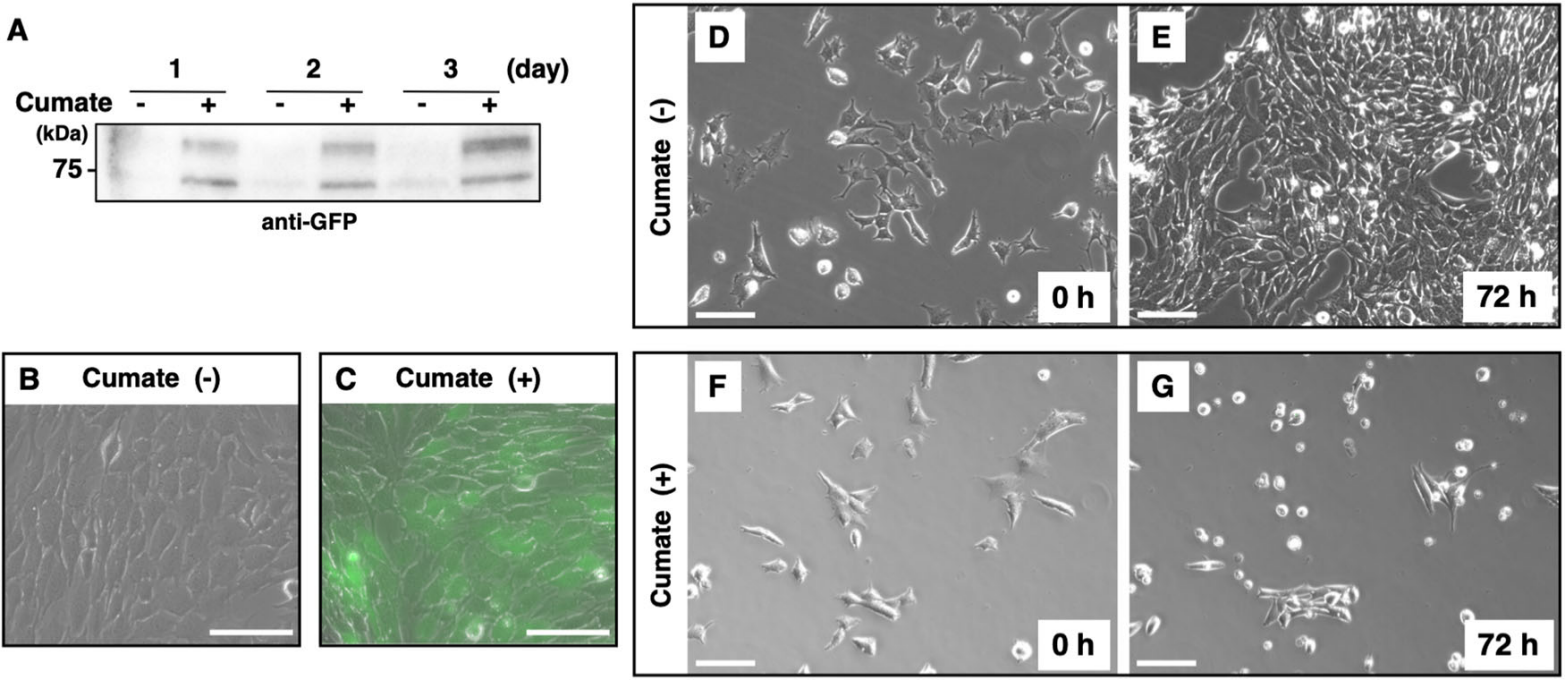
Effects of drug-induced expression of GALNTL5 protein in GC-2spd(ts) cells. **A** Western blotting with anti-GFP antibody. In the GC-2spd(ts) cell line with a chemically inducible expression vector for GALNTL5 tagged with GFP (GALNTL5-GFP/GC-2spd), GALNTL5 protein was detected as at least two bands for 3 days when the cumate chemical solution was present. **B**, **C** Fluorescence microscopy image of GALNTL5-GFP/GC-2spd cells after 24 h with or without chemical induction. White scale bars, 100 μm. **D**, **E** GALNTL5-GFP/GC-2spd cells at baseline and after 72 h, without chemical induction. Live-cell imaging of cell proliferation was performed with an All-in-one Fluorescence Microscope (Keyence). **F**, **G** Cell imaging of GALNTL5-GFP/GC-2spd at baseline and after 72 h of chemical induction. At 72 h there is continuous GALNTL5-GFP expression and shrinking cells are seen. White scale bars, 100 µm. Detailed cellular alterations with or without chemical induction are shown in the supplementary movies.

No stable cell lines have been successfully transfected with the mouse *Galntl5* gene. This strongly suggests that GALNTL5 is toxic to all cultured cells except for differentiating spermatids. To investigate the effect of GALNTL5 in vitro, we decided to establish cell lines in which GALNTL5 expression was chemically induced. To this end, we employed the GC-2spd(ts) cell line because it is derived from mouse spermatocytes, which do not express *Galntl5* mRNA. Using reverse transcription-polymerase chain reaction (RT-PCR), we confirmed that the GC-2spd(ts) cell line does not express *Galntl5* mRNA (Supplementary Fig. 1A). We consequently succeeded in establishing two independent GC-2spd(ts) cell lines that expressed drug-inducible GALNTL5 protein tagged with green fluorescent protein (GFP) (GALNTL5-GFP/GC-2spd). Without chemical induction, the signal of GALNTL5 protein tagged with GFP was hardly observable with either western blotting using an anti-GFP antibody (minus lanes in Fig. 1A) or with fluorescence microscopy (Fig. 1B). In contrast, GALNTL5 protein was continuously expressed in GALNTL5-GFP/GC-2spd cells for 1–3 days when cumate was added (+ lanes in Fig. 1A). GALNTL5-GFP signals in live cells were captured with fluorescence microscopy after 1-day culture following the addition of cumate solution (Fig. 1C).

### GALNTL5 expression is cytotoxic and results in apoptosis with cell shrinkage

Regarding cell proliferation, continuous GALNTL5 expression suppressed the number of cells approximately 1.5-fold after 72 h of culture (cumate (+), Supplementary Fig. 1B). The MTS assay also showed that continuous GALNTL5 expression suppressed cell growth (Supplementary Fig. 1C), whereas GALNTL5-GFP/GC-2spd cells proliferated normally without chemical induction for 3 days (Fig. 1D, E and Supplementary Movie 1). However, the continuous expression of GALNTL5 tagged with GFP resulted in cell death with shrinkage during the 3 days in which cumate was added (Fig. 1F, G and Supplementary Movie 2). Cells exhibiting cumate-induced GALNTL5-GFP signals contracted with the appearance of a deflating balloon (Supplementary Movie 3). Cumate exposure itself had no effect on the proliferation of normal GC-2spd(ts) cells (Supplementary Movie 4). The cell death caused by GALNTL5 was not mediated by the autophagy system, because continuous expression of GALNTL5 apparently decreased the expression of LC3, a marker of autophagy that reflects intracellular autophagic activity (Supplementary Fig. 1D, E). Flow cytometry analysis of Annexin V expression showed remarkable increase in the rate of apoptosis in GALNTL5-GFP/GC-2spd cells during GALNTL5 expression (cumate (+) column, Supplementary Fig. 1F). These data allowed us to demonstrate, for the first time, that GALNTL5 is cytotoxic to cultured cells and that it induces apoptotic cell death.

### GALNTL5 co-localizes in the endoplasmic reticulum (ER) with its chaperone ER-resident proteins

**Fig. 2:**
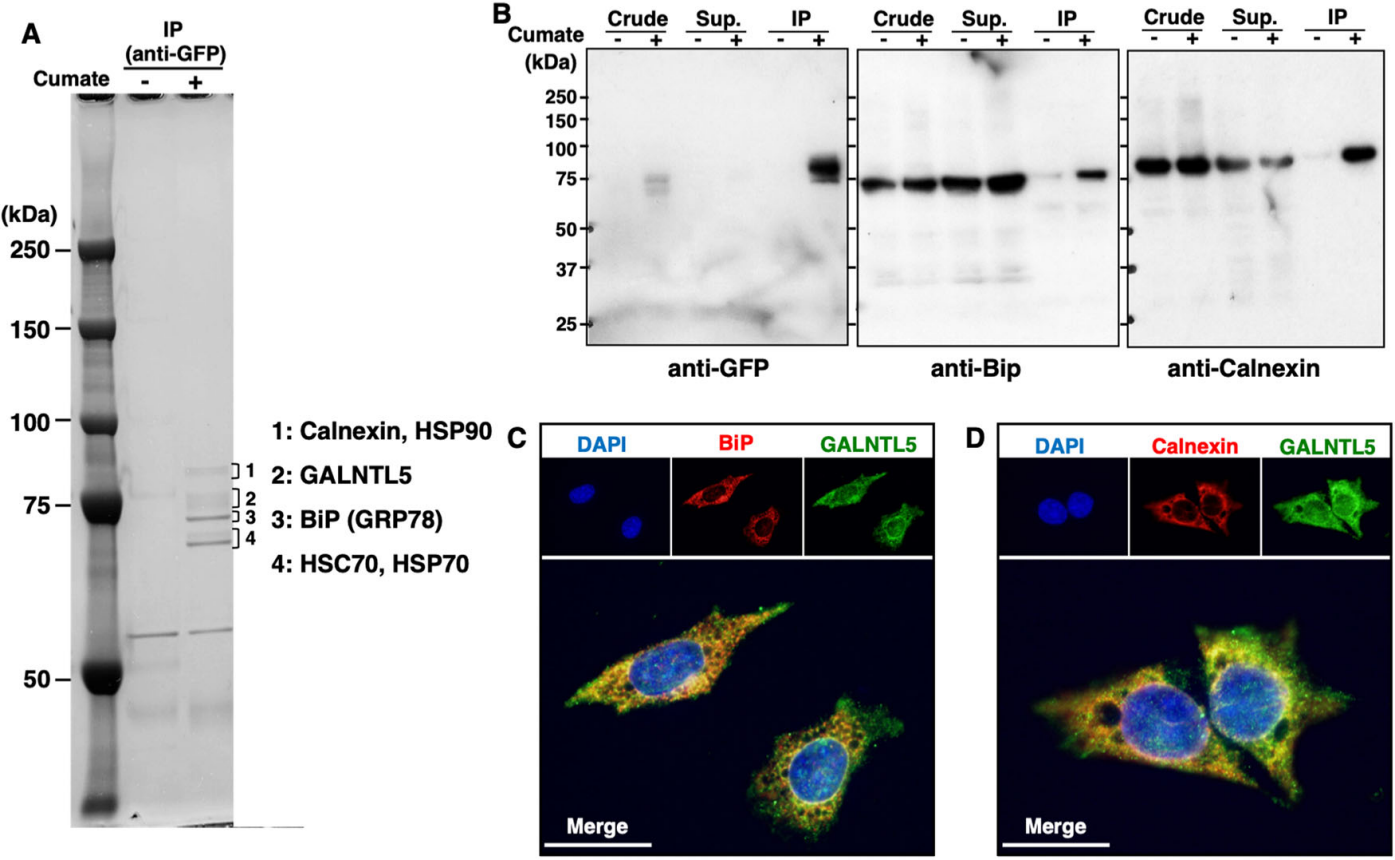
The direct interaction of GALNTL5 with ER-resident chaperone proteins. **A** Co-immunoprecipitants with anti-GFP antibody were separated on SDS-PAGE and visualized using the Negative Gel Stain MS Kit (Fujifilm Wako Pure Chemical Corporation). From the four fractions of visualized bands around 75 kDa, five chaperone proteins (calnexin, HSP90, BiP, HSC70, and HSP70) were identified with MS. **B** Anti-BiP and anti-calnexin antibodies confirmed that BiP and calnexin interact with GALNTL5. **C**, **D** Fluorescence image of GALNTL5-GFP/GC-2spd cells stained with anti-BiP antibodies, anti-calnexin antibodies, or DAPI nuclear staining, after 1 day in the presence of cumate solution. Yellow signals represent GALNTL5-GFP, and BiP or calnexin co-localization in the ER. White scale bars, 20 µm.

**Fig. 3:**
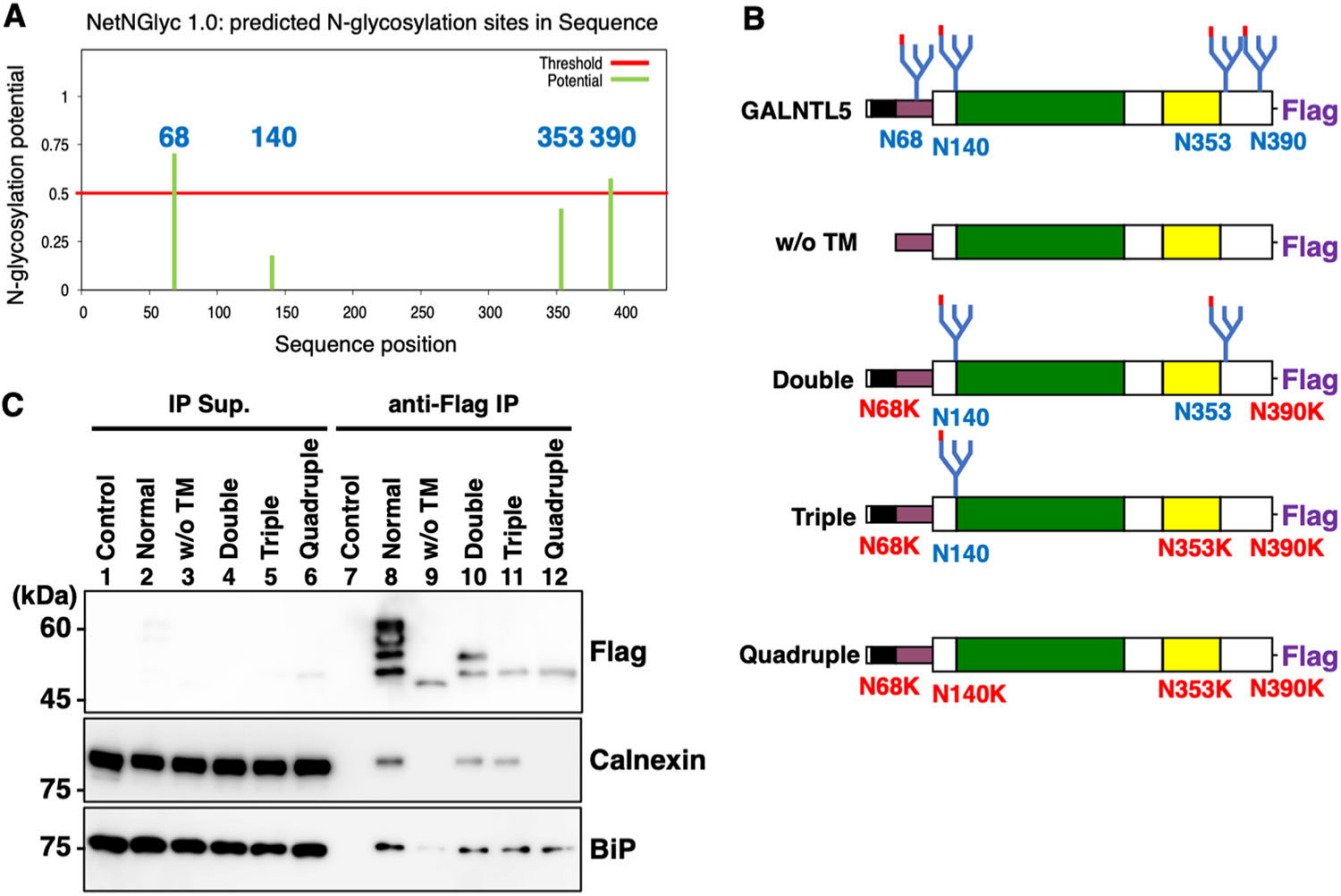
Interaction of GALNTL5 with the ER-resident proteins calnexin and BiP, the former through ER *N*-glycosylation. **A** A search program for *N*-glycosylation sites (https://services.healthtech.dtu.dk/services/NetNGlyc-1.0/) predicted that four asparagine residues (68, 140, 353, and 390) in the amino acid sequence of mouse GALNTL5 were N-glycosylation sites. **B** Schematic comparison of mouse GALNTL5 based on IP experiments. Full-length GALNTL5 contains a transmembrane domain (back), a stem region (purple), and a catalytic unit consisting of a GT1 motif (green), a Gal/GalNAc-T motif (yellow), and a Flag tag at the C-terminus. N68, N140, N353, and N390 indicate asparagine residues that serve as *N*-glycosylation sites in mouse GALNTL5. “w/o TM” indicates full-length GALNTL5 without the transmembrane domain. The double mutants are characterized by substitution of asparagine for lysine at two amino acid sites (N68K and N390K). The triple mutants show substitution of asparagine for lysine at three amino acid sites (N68K, N353K, and N390K). The quadruple mutants exhibit lysine at all sites (N68K, N140K, N353K, and N390K). **C** IP with anti-Flag antibody followed by western blotting with anti-Flag antibody. Co-IP with anti-Flag antibody followed by western blotting with anti-calnexin and anti-BiP antibodies.

GALNTL5 possesses a catalytic unit and also the transmembrane domain common to the *N*-acetyl-galactosaminyl-transferase gene family, but not the lectin domain (Fig. 3B) [6]. Thus far, no molecules have been identified that directly interact with the GALNTL5 protein. To estimate the cytotoxic effects of GALNTL5, we sought to detect molecules that interact with GALNTL5 by performing immunoprecipitation (IP) experiments using anti-GFP antibodies. Several bands around 75 kDa were observed on SDS-polyacrylamide gel electrophoresis (SDS-PAGE). Mass spectrometry (MS) analysis showed that these bands represented various chaperone proteins (Fig. 2A) [10, 11]. IP and western blotting confirmed the direct interaction between GALNTL5 and the ER-resident chaperone proteins calnexin and BiP (Fig. 2B, Supplementary Fig. 2A). GALNTL5 did not directly interact with other chaperone molecules identified in MS analyses (Supplementary Fig. 2A). Immunofluorescence microscopy showed that GALNTL5 co-localized independently with calnexin and BiP in GALNTL5-GFP/GC-2spd cells (Fig. 2C, D). These data strongly suggest that GALNTL5 retains in ER interacts with calnexin and BiP, two ER-resident chaperone proteins.

### The ER-resident proteins calnexin and BiP bind to GALNTL5, and calnexin specifically recognizes and binds to its *N*-glycan structures

To confirm the interaction between GALNTL5 and the chaperone proteins identified in MS analyses, we performed IP experiments in which GC-2spd(ts) cells were transiently transfected with Flag-tagged mouse GALNTL5 cDNA (Supplementary Fig. 2A). In this IP / western blot experiment, the antibody against the Flag tag detected four GALNTL5 protein bands as IP products (Flag panel in Supplementary Fig. 2A). In contrast transient expression of GALNTL5 cDNA with removal of the transmembrane domain produced a single GALNTL5 protein band (Supplementary Fig. 2B, C). Furthermore, GALNTL5 protein without the transmembrane was distributed in the cytoplasm and did not co-localize with calnexin (Supplementary Fig. 2D). It is possible that the lack of the N-terminus may have inhibited the transport of GALNTL5 to the ER. Our data at least suggest that the N-terminus of GALNTL5, including the transmembrane domain, work to tether GALNTL5 to the ER and contributes to its protein modification within the ER. In fact, IP/western blotting confirmed that the transmembrane domain of GALNTL5 is essential for the interaction between GALNTL5 and calnexin (lane 9 in Fig. 3C).

Given that GALNTL5 resides in the ER and interacts with calnexin, we predicted that GALNTL5 modification would occur via *N*-linked glycosylation [12]. Using a search program for *N*-glycosylation sites (https://services.healthtech.dtu.dk/services/NetNGlyc-1.0/) [13], we identified four candidate positions of the mouse GALNTL5 protein (Fig. 3A). We constructed cDNAs of mouse GALNTL5 with mutations at the sites most likely to be *N*-glycosylated, and examined whether or not the resulting proteins interacted with calnexin (Fig. 3B). Calnexin did not interact with non-*N*-glycosylated GALNTL5 (lane 12 in Fig. 3C), whereas BiP bound to GALNTL5 with or without *N*-glycosylation modification, and very weakly to GALNTL5 lacking the transmembrane domain (BiP panel in Fig. 3C). This indicates that GALNTL5 binding to calnexin or BiP requires the retention of GALNTL5 in the ER via its transmembrane domain, and in particular, that the *N*-glycosylation of GALNTL5 is necessary for its interaction with calnexin. Experiments using HEK293T cells similarly confirmed that the transmembrane domain of the human GALNTL5 protein was required for binding to calnexin and BiP, and calnexin interacted with this protein through its *N*-glycan structures (Supplementary Fig. 3).

### GALNTL5 impairs the binding of calnexin to unfolded proteins

As a chaperone, calnexin assists in protein folding and quality control, ensuring that only properly folded and assembled proteins progress further along the secretory pathway [14]. Specifically, it acts to retain unfolded or unassembled *N*-linked glycoproteins in the ER [12]. Glycoproteins that cannot be correctly folded by the calnexin cycle are exported to the endoplasmic reticulum-associated protein degradation (ERAD) system through BiP [15]. Calnexin associates with mutant unfolded glycoproteins during quality control. It has been reported that null Hong Kong (NHK), a variant of the glycoprotein α1- antitrypsin with 61 truncated amino acids at the C-terminal, directly interacts with calnexin [16].

**Fig. 4:**
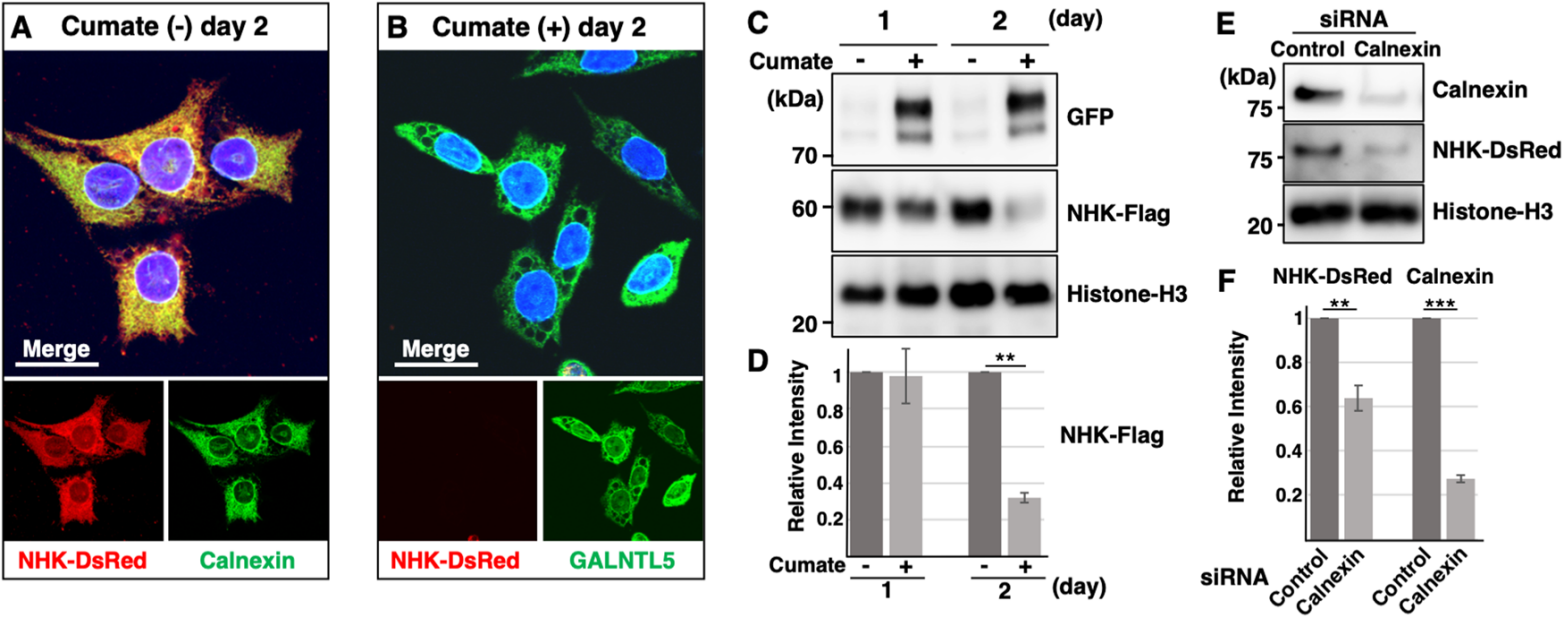
GALNTL5 localization to the ER impairs calnexin function. **A**, **B** Fluorescent images of GALNTL5-GFP/GC-2spd cells stably expressing NHK tagged with DsRed2. Without the induction of GALNTL5-GFP expression, the NHK-DsRed signals co-localize with calnexin in the ER (**A**). When the expression of GALNTL5-GFP was induced with a cumate solution, NHK-DsRed signals disappeared from the ER 2 days later but those of GALNTL5-GFP persisted (**B**). Nuclei are stained with DAPI. White scale bars in the merged image, 20 µm. **C** Transient expression of NHK tagged with Flag in GALNTL5-GFP/GC-2spd cells. NHK-Flag signals decreased with persistent, 2-day GALNTL5 expression in GALNTL5-GFP/GC-2spd cells. Histone-H3 was used as the control. **D** Quantification of band intensity of NHK-Flag signals were statistically analyzed (mean ± SEM. *n* = 3. Two-tailed Student’s *t*-test. **p < 0.01). **E** Western blot of calnexin and NHK-DsRed (NHK protein tagged with DsRed) in GC-2spd(ts) cells expressing NHK-DsRed at 72 h after calnexin siRNA induction. Histone-H3 was used as the control. **F** The alterations of protein levels of calnexin and NHK-DsRed were quantified (mean ± SEM. *n* = 3. Two-tailed Student’s *t*-test. **p < 0.01, ***p < 0.001).

To determine if GALNTL5 affects the interaction between calnexin and unfolded proteins, we used GALNTL5-GFP/GC-2spd to establish a stable cell line expressing NHK tagged with *Discosoma sp*. Red Fluorescent Protein (DsRed2). Without the induction of GALNTL5-GFP expression, NHK-DsRed signals co-localized with calnexin, confirming that calnexin directly interacts with the unfolded glycoproteins in the ER (Fig. 4A). However, after chemical induction of GALNTL5-GFP for 2 days, NHK-DsRed signals in the ER disappeared (Fig. 4B). By transiently transfecting GALNTL5-GFP/GC-2spd cells with Flag-tagged NHK, we examined alterations in NHK proteins with or without GALNTL5-GFP expression. On day 2 under GALNTL5-GFP expression, NHK protein degradation was detected with western blot analysis (Fig. 4C, D). Knockdown using siRNA specific to calnexin confirmed that the impairment of calnexin function caused the NHK protein degradation (Fig. 4E, F). These data indicate that GALNTL5 blocks calnexin function in the calnexin cycle, thereby promoting the degradation of unfolded proteins that would otherwise have been retained in the ER by calnexin.

### The appearance of GALNTL5 enhances the degradation of proteins responsible for maintaining cell function

To determine how cells are affected by the interaction between GALNTL5 and calnexin in the ER, we newly established two GC-2spd(ts) cell lines, one that expressed drug-inducible GALNTL5 protein tagged with Flag (GALNTL5-Flag/GC-2spd), and the other that expressed GALNTL5 carrying quadruple mutants at four *N*-glycosylation sites (see in Fig. 3B, GALNTL5Quad-Flag/GC-2spd). A cell counting assay and MTS assay showed that the proliferation of both cell lines was suppressed by about half after 3-day induction with cumate (Supplementary Fig. 4A, B). The stable expression of *N*-glycosylated GALNTL5 was again confirmed to induce the apoptosis (Supplementary Fig. 4D). Continuous expression of non-glycosylated GALNTL5 also induced apoptosis of GALNTL5Quad-Flag/GC-2spd, but the progression of apoptosis was slower than in GALNTL5-Flag/GC-2spd (Supplementary Figure 4E). Meanwhile, NHK protein degradation progressed in GALNTL5-Flag/GC-2spd expressing *N*-glycosylated GALNTL5 with cumate, but not in GALNTL5Quad-Flag/GC-2spd expressing non-glycosylated GALNTL5 (Supplementary Fig. 5). These data indicated that continuous expression of *N*-glycosylated or non-glycosylated GALNTL5 elicits cell death, but that only *N*-glycosylated GALNTL5 specific binding to calnexin dissociates calnexin from its interaction with the NHK protein. The slow apoptotic progression of GALNTL5Quad-Flag/GC-2spd may be influenced by the binding of BiP to non-glycosylated GALNTL5. As shown below, the binding of BiP to non-glycosylated GALNTL5 may also be somewhat responsible for the mild reduction of proteins involved in cell maintenance.

**Fig. 5:**
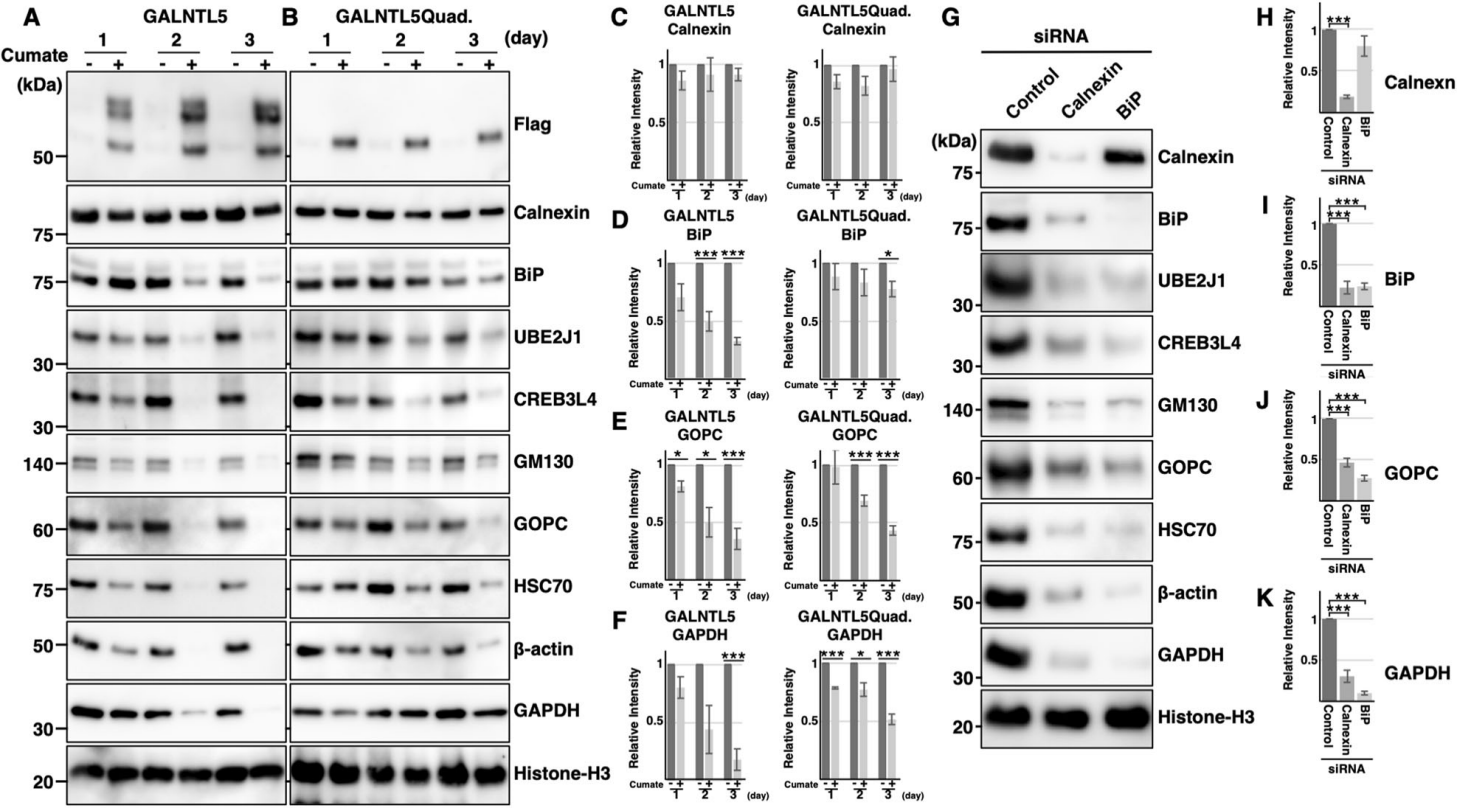
Influence of persistent GALNTL5 expression on GC-2spd(ts) cells. **A** Continuous culture of GALNTL5-Flag/GC-2spd cells with or without cumate solution for 3 days. The N-glycosylated products of GALNTL5-Flag were detected for 3 days in lanes indicating chemical induction (+). Signal intensities of calnexin and of histone H3 nuclear protein as a control were almost identical at 3 days, with or without chemical induction. With accumulation of GALNTL5 in GALNTL5-Flag/GC-2spd cells, the levels of three ER proteins (BiP, UBE2J1, and CREB3L4), Golgi proteins, (GM130 and GOPC), and cytoplasmic proteins (HSC70, β-actin, and GAPDH) were hardly detected by western blotting after 3 days. **B** Continuous culture of GALNTL5Quad-Flag/GC-2spd cells with or without cumate solution for 3 days. In GALNTL5Quad-Flag/GC-2spd cells, signals of calnexin or of histone H3 as a control were also detected uniformly for 3 days, with or without chemical induction. The levels of the three ER marker proteins, Golgi markers, and cytoplasmic markers were slightly decreased after induction for 3 days. Histograms representing signal intensities detected with anti-calnexin antibody (**C**), anti-BiP antibody (**D**), anti-GOPC antibody (**E**), or anti-GAPDH antibody (**F**), in continuous culture of GALNTL5-Flag/GC-2spd cells and GALNTL5Quad-Flag/GC-2spd cells with or without cumate solution for 3 days. All values are means ± SEM (error bars, *n* = 3). Two-tailed Student’s t-test with or without cumate treatment. *p < 0.05, ***p < 0.001. **G** siRNA knockdown of calnexin or BiP in GC-2spd(ts) cells. Knockdown with calnexin-specific siRNA decreased the amount of BiP protein (BiP panel in the calnexin lane). Knockdown of BiP had almost no effect on calnexin levels (calnexin panel in the BiP lane). Knockdown of both calnexin and BiP decreased the expression of component proteins in the ER, Golgi apparatus, and cytoplasm. Histone H3 was used as the control. Quantification of band intensities detected with anti-calnexin (**H**), anti-BiP (**I**), anti-GOPC (**J**), and anti-GAPDH (**K**) antibodies in three categories of GC-2spd(ts) cells: 72-h transfection with control, calnexin or BiP siRNA. The error bars are presented as mean ± SEM (*n* = 3). ***p < 0.001.

We next compared how protein compositions were altered by the continuous expression of either *N*-glycosylated or non-glycosylated GALNTL5 (Flag panel in Fig. 5A, B). Interestingly, during 3-day culture of both cell lines after the induced expression of *N*-glycosylated or non-glycosylated GALNTL5, calnexin levels showed hardly any effect (Calnexin panel in Fig. 5A–C). However, the level of BiP significantly decreased with *N*-glycosylated GALNTL5 protein but not with the quadruplet mutant of GALNTL5 (BiP panel in Fig. 5A, B, D). These results strongly suggest that the calnexin dysfunction caused by the interaction between calnexin and GALNTL5 lead to decreased BiP expression. The attenuation of BiP was reproduced by calnexin knockdown using calnexin-specific siRNA (BiP panels in Fig. 5G, I). Taken together, these results show that GALNTL5 binding to calnexin impair calnexin function and consequently suppress BiP expression.

In western blots, the signal intensities of other ER constituent proteins, namely UBE2J1 [17], CREB3L4 [18], IRE1α [19] and OS9 [20], were clearly decreased in both cell lines, though the magnitudes of the reductions were smaller in GALNTL5Quad-Flag/GC-2spd than in GALNTL5-Flag/GC-2spd (UBE2J1 and CREB3L4 panels in Fig. 5A, B, Supplementary Fig. 6A, B and Supplementary Fig. 7A, B). A similar pattern of decreased expression was observed for two Golgi-associated proteins [3], GM130 and GOPC, as well as the cytoplasmic proteins HSC70, β-actin, GAPDH, and HSP-70 (corresponding panels in Fig. 5A, B, E, F and Supplementary Fig. 6A, B and Supplementary Fig. 7A, B). These data suggest that the interaction of calnexin with GALNTL5 leads to severe cellular damage, such as apoptosis, by causing a decrease in proteins involved in cell maintenance. In contrast, non-glycosylated GALNTL5, which binds to BiP but not to calnexin, induced apoptosis and reduced the expression of proteins that maintain cell activity, but its effect was weaker than that of normal GALNTL5.

Once again, to confirm the alterations in the amounts of proteins involved in cell maintenance due to the dysfunction of calnexin or BiP, we conducted knockdown experiments using the respective siRNAs in normal GC-2spd cells. As expected, siRNAs against both calnexin and BiP reduced the protein components of the ER, Golgi apparatus, and cytoplasm (Fig. 5G–K, Supplementary Fig. 6C and Supplementary Fig. 7C). Interestingly, although siRNA knockdown of BiP had no significant effect on the amount of calnexin in GC-2spd cells (Fig. 5H), siRNA knockdown of calnexin significantly reduced the expression of BiP, as mentioned above (Fig. 5I). This is the first report showing that calnexin knockdown reduces the expression of other protein components by causing a decrease in BiP. Taken altogether, our data indicate that GALNTL5 binds strongly to calnexin in the ER, causing calnexin dysfunction and impairing BiP expression, thus resulting in cell death (Supplementary Fig. 6D).

### The effects of GALNTL5 on cell structure

**Fig. 6:**
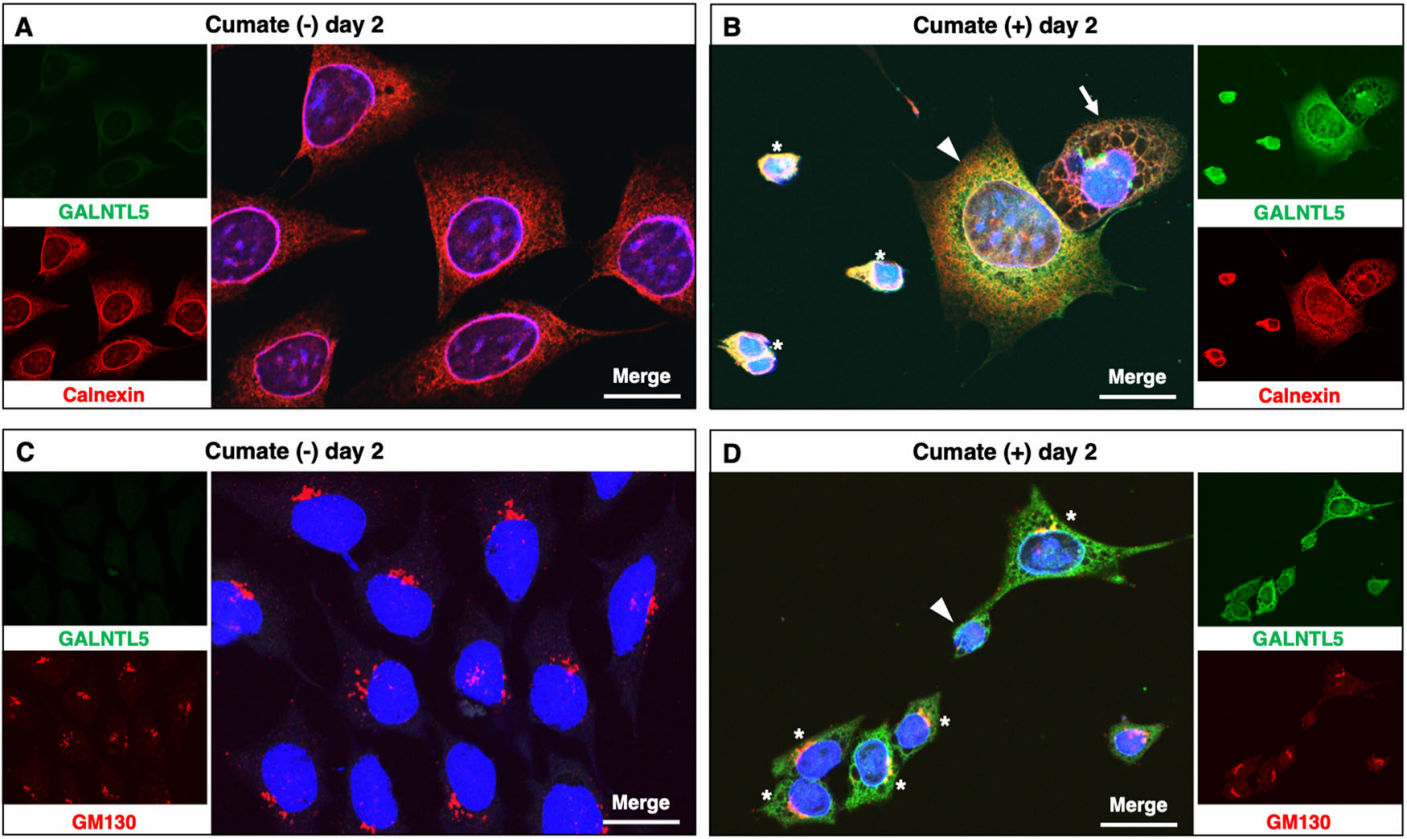
Influence of persistent GALNTL5 protein expression on cell structure. **A** Fluorescence image of GALNTL5-GFP/GC-2spd cells treated with anti-calnexin antibody and without cumate induction. **B** Fluorescence image of GALNTL5-GFP/GC-2spd cells treated with anti-calnexin antibody after 2 days in the presence of cumate solution. In the cell marked with the white arrowhead, the ER is stained with anti-calnexin antibody and it extends widely throughout the cytoplasm. In the cell marked with the white arrow, the ER is enlarged and has a rough mesh structure. The cells marked with white asterisks show constriction of nuclei, and calnexin and GALNTL5 signals are seen around the nuclei. **C** Fluorescent image of GALNTL5-GFP/GC-2spd cells with anti-GM130 antibodies staining, and without cumate treatment. **D** Fluorescent image of GALNTL5-GFP/GC-2spd cells with anti-GM130 antibody after 2 days in the presence of cumate solution. In the cells marked with white asterisks, GM130 signals have accumulated on the nuclear membrane. In the cell with a smaller nucleus (white arrow), GM130 signals are barely visible. In each merged image, the nucleus was stained with DAPI. White scale bars, 20 µm.

In the absence of GALNTL5 expression, signals from ER-resident calnexin showed localization around the nucleus and distribution throughout the cytoplasm in a fine mesh pattern (Fig. 6A). In contrast, 2 days after the induction of GALNTL5-GFP expression, various alterations in cell morphology were observed by immunofluorescence microscopy (Fig. 6B). The co-localization of GALNTL5-GFP and calnexin was more widespread in the cytoplasm (white arrowhead in Fig. 6B) than in a normal cell. Enlargement of the ER structure in a rough mesh pattern was also observed (white arrow in Fig. 6B). Figure 2C, D and Fig. 4B also show that GALNTL5-GFP was distributed in the cytoplasm in a large, prominent reticulate pattern. Finally, the co-localized calnexin and GALNTL5 signals led to a constricted nuclear appearance (white asterisks in Fig. 6B). These images suggested that the accumulation of GALNTL5 in the ER changes the ER conformation, and that the ER membrane, containing both GALNTL5 and calnexin, causes nuclear contraction.

Next, using anti-GM130 antibody as a *cis*-Golgi marker, we tried to observe alterations in the Golgi apparatus. In the absence of GALNTL5 expression, the *cis*-Golgi apparatus appeared as a ribbon-like structure in the perinuclear region (Fig. 6C) [21]. When GALNTL5-GFP expression was induced for 2 days, GM130 signals were restricted to the nuclear membrane (white asterisks in Fig. 6B) or were hardly detected in cells with smaller nuclei (white arrow in Fig. 6D). These data support the hypothesis that the co-localization of GALNTL5 and calnexin in the ER induces conformational changes in the Golgi apparatus.

## Discussion

In this study, we established a drug-induced expression system that enabled sustained expression of GALNTL5 in GC-2spd(ts) cells. This is the first demonstration that persistently expressed GALNTL5 is retained in the ER and binds directly to the ER-resident chaperone proteins calnexin and BiP, resulting in disturbed ER homeostasis that impedes the supply of functional proteins and causes cell death by apoptosis with cell shrinkage. Our analysis strongly suggests that the impairment of ER function by GALNTL5 is involved in ER regression in differentiating spermatids [22, 23].

Morphological alterations in differentiating spermatids have been visualized using electron microscopy [22, 24], but the underlying mechanism is unclear. Our experiments showed that the induced expression of GALNTL5 resulted in a thicker and rougher network of ER tubular structures apart from the nucleus. It was also reported that the loss of BiP function expanded the ER lumen [25, 26], and our results in terms of changes in ER structure are consistent with those observed in the dysfunction of BiP due to calnexin binding to GALNTL5. Moreover, in the cytoplasm of the posterior region far from the spermatid nucleus in the maturation phase, the diameters of abundant ER tubules were observed to be thicker than those of intracellular tubules [22]. Taken together, these data support the possibility that during spermatid morphogenesis, GALNTL5 is a resident component of the ER that contributes to altering the ER tubular structure distal to the nucleus by attenuating ER function.

Regarding ER-resident proteins, including GRP94 (an endoplasmic chaperone), RNF133 (a testis-specific E3 ubiquitin ligase that directly interacts with UBE2J1), CREB3L4, and UBE2J1, mice deficient for each corresponding gene exhibited deformed sperm heads as a common phenotype [17, 27–29]. It is interesting to note that the molecules constituting the functional ER are responsible for canonical sperm head formation. Our experiments showed that the sustainable localization of GALNTL5 in the perinuclear ER, in concert with calnexin, resulted in nuclear compaction. This indicates that the calnexin dysfunction caused by GALNTL5 may result in nuclear condensation of the spermatid head by preventing maintenance of the ER structure.

Our study showed that GALNTL5 retained in the ER impairs the function of calnexin and BiP, which are chaperone proteins responsible for protein folding, quality control, and secretion. Therefore, depletion of the proteins secreted from the ER to the Golgi apparatus would also affect Golgi apparatus maintenance. Indeed, GALNTL5 expression decreased the expression of protein components of the Golgi apparatus and condensed signals of the *cis*-Golgi marker, GM130, in the perinuclear area. In mice deficient for genes encoding protein components of the Golgi apparatus [30–32], the acrosome is thought to be derived from the Golgi apparatus [3]. The blockage of protein secretion into the Golgi apparatus by ER-resident GALNTL5 could be involved in the biosynthesis of Golgi-derived acrosomes.

The ER becomes a source of autophagic membranes when its homeostasis is disrupted by stress or other factors [33, 34]. Recently, studies involving mice deficient in autophagy-related genes revealed that autophagic machinery contributes to spermatid differentiation [35]. It is quite possible that GALNTL5 expression is also involved in supplying many autophagic membranes by acting as a trigger for ER stress in sperm differentiation. A more precise system regulating GALNTL5 expression in the near future would elucidate the more exact function of it in spermiogenesis. Moreover, if it is possible to establish to specifically express GALNTL5 in cancer cells, GALNTL5 might serve as a novel anti-cancer drug.

## Materials and methods

### Plasmid DNA constructs

Mouse *Galntl5* cDNA [6] was subcloned into the pAcGFP-N1 vector (Clontech) to add the GFP tag, or into the pCMV6-Entry vector (OriGene) to add the Flag tag. To construct mouse *Galntl5* cDNA with a truncated transmembrane domain, DNA was amplified with polymerase chain reaction (PCR) using the following primers: 5′-ATGCTTGAACAAGAAGACATGC-3′ and 5′-GAAACGATTTTTTTTCCTTTTCCTCTCTGTGTTAAATGG-3′. Human *GALNTL5* cDNA (#MHS6278-202807229) was purchased from Dharmacon^TM^ and subcloned into the pCMV6-Entry vector to add the Flag tag. PCR of human *GALNTL5* cDNA with a truncated transmembrane domain used the following primers: 5′-ATGCATAATCATGTGAGCAGCTGG-3′ and 5′-CAGGCTGTTCACAGATGC-3′. To construct a model ERAD substrate, cDNAs of NHK, human α1-antitrypsin with 61 truncated amino acids at the C-terminal, and human SERPINA1 (#MHS6278-202756262) were also purchased from Dharmacon^TM^. The fragment of truncated human α1-antitrypsin was amplified with PCR using the following primers: 5′-ATGCCGTCTTCTGTCTCG-3′ and 5′-CACGGCCTTGGAGAGCTTCAGG-3′; it was then subcloned into the pCMV6-Entry vector or the pDsRed2-N1 vector (Clontech).

### Site-directed mutagenesis

The primer set designed to introduce mutations into mouse *Galntl5* and human *GALNTL5* is shown in Supplementary Table 1. All mutations were generated using the PrimeSTAR mutagenesis basal kit (Takara Bio). After desired mutations were confirmed with DNA sequencing, the mutant cDNAs of the GALNTL5 protein were transfected into GC-2spd(ts) cells or 293 T cells using Avalanche-Omni Transfection Reagent (EZ Biosystems, College Park, MD, USA).

### RT-PCR

Total RNA was isolated from GC-2spd(ts) cells using the RNeasy Mini kit (QIAGEN, Inc., Valencia, CA, USA). A reverse transcriptase reaction with 1 μg of total RNA was carried out with a first-strand cDNA synthesis kit (ReverTra Ace α; TOYOBO Co., Ltd, Osaka, Japan). Thereafter, 1-μl aliquots of the RT reaction products were used for PCR to amplify GALNTL5 mRNA. The following sets of oligonucleotide primers were used: mGALNTL5F, 5′-TGGCGGCACCTATTGTAAGG-3′; mGALNTL5R, 5′-GGAATGACTTGCAACCCAGG-3′. Amplification was performed using Taq polymerase (PerkinElmer, Waltham, MA, USA) over 35 cycles. Each cycle consisted of denaturation at 94°C for 1 min, annealing at 54°C for 1 min, and extension at 72°C for 1 min. The expression levels determined by RT-PCR were compared with expression levels in mouse testis as a positive control.

### Cell culture

The GC-2spd(ts) cell line was purchased from American Type Culture Collection (CRL-2196) and cultured at 37 °C under 5% CO_2_ in Dulbecco’s modified Eagle’s medium (DMEM, Fujifilm Wako Pure Chemical Corporation) supplemented with 10% fetal bovine serum (FBS, Gibco), 100 IU/ml of penicillin, and 100 µg/ml of streptomycin. 293T cells were also maintained in DMEM containing 10% FBS and 100 IU/ml of penicillin-streptomycin at 37°C under 5% CO_2_.

### Establishment of GALNTL5-GFP/GC-2spd(ts), GALNTL5-Flag/GC-2spd(ts), and quadruple GALNTL5-Flag/GC-2spd(ts) cell lines, all expressing tagged GALNTL5 via chemical induction

To establish the GC-2spd(ts) cell line expressing GALNTL5, *GALNTL5* cDNA tagged with GFP or Flag was subcloned into the Enhanced Episomal Vectors cloning and expression vector format, which features an ultra-tight cumate inducible system (catalog #EEV61A-1, System Biosciences, Palo Alto, CA, USA). Avalanche-Omni Transfection Reagent (EZ Biosystems) was employed as the DNA transfection reagent for the cell lines. GC-2spd(ts) cells containing the transfected DNA were screened and maintained with 8 mg/ml of puromycin (InvivoGen, Carlsbad, CA, USA).

### Live-cell imaging

GALNTL5-GFP/GC-2spd(ts) cells were seeded in 12-well plates at a cell density of 2~4 × 10^4^ cells and incubated for 24 h before cumate was added. Live-cell imaging was performed using a humidified stage-top chamber (STR Stage Top Incubator, TOKAI HIT, Japan) that maintained the cells at 37°C under 5% CO_2_. The chamber was mounted to a BZ-X800 All-in-one Fluorescence Microscope (Keyence, Osaka, Japan). An image at each *xy* coordinate was taken every 15 to 20 minutes for 0–72 h. For each coordinate, images were captured for phase contrast and the GFP fluorescent filter using a live-cell imaging system (BZ-X800 Viewer, Keyence, Japan), and analyzed using the BZ-X800 Analyzer (Keyence) to produce a video over the period of incubation.

### Apoptosis assay

To examine changes in apoptosis rates associated with GALNTL5 expression in GC-2spd(ts) cells, GALNTL5-GFP/GC-2spd, GALNTL5-Flag/GC-2spd, and GALNTL5Quad-Flag/GC-2spd cells were treated with cumate solution for 3 days. As a negative control, cells from each cell line cultured without cumate solution were used. Apoptosis was measured based on fluorochrome-conjugated Annexin V staining using an APC Annexin V Apoptosis Detection Kit (BioLegend, San Diego, CA, USA) and flow cytometry.

### Cell count experiments

GALNTL5-GFP/GC-2spd, GALNTL5-Flag/GC-2spd, and GALNTL5Quad-Flag/GC-2spd cells were plated at 4 × 10^4^ in each well of 24-well plates with or without cumate solution for 72 h. In both cases, the number of cells was counted every 24 h.

### MTS assay

Cells were seeded into a 96-well plate at a density of 4000 cells per well for 72 h. Cell viability was measured after 0, 24, 48, and 72 h using 3-(4,5-dimethylthiazol-2-yl)-5-(3-carboxymethoxyphenyl)-2-(4-sulfophenyl)-2H-tetrazolium inner salt (MTS) (Promega Corporation, Madison, WI, USA). Absorbance was measured at a wavelength of 490 nm with a Viento 808 IU absorbance reader (BioTek, Winooski, VT, USA).

### Statistical analysis

All experimental data are presented as means ± SEM. Student’s *t*-test was used to compare means between two groups. All statistical analyses were performed using GraphPad Prism8 software (GraphPad Software Inc., San Diego, CA, USA). *P* < 0.05 was considered statistically significant.

### Western blot analysis

GALNTL5-GFP/GC-2spd(ts) and GALNTL5-Flag/GC-2spd(ts) cell lines were cultured in DMEM with 10% FBS for 24 h. The cells were then treated in DMEM with or without 0.1% cumate solution (QM150A-1, System Biosciences) for 1–3 days. After treatment, cells lysates were resolved in SDS-PAGE and transferred onto an Immune-Blot PVDF membrane (Bio-Rad, Richmond, CA, USA) for immunoblotting. Membranes were immunoblotted with antibodies and appropriate buffers as listed in Supplementary Table 2.

### siRNA knockdown of calnexin and BiP

For siRNA experiments, DharmaFECT 1 Transfection Reagent (T-2001-01, Dharmacon^TM^) was used to transfect GC-2spd(ts) cells for 72 h with 5 μM of mouse calnexin siRNA SMARTpool (M-056876-0005, Dharmacon^TM^), mouse BiP5 siRNA [36], or siGENOME non-targeting siRNA as a negative control (D-001206-13-05, Dharmacon^TM^). The whole-cell extracts were obtained for western blot analysis as described above.

### IP

To identify molecules that interacted with GALNTL5, GALNTL5-GFP/GC-2spd(ts) cells were cultured for 2 days in DMEM medium with cumate solution. The cell lysates were used for IP, which was carried out using anti-GFP mAb-Magnetic Beads (MBL, Japan). The purification products were loaded on an SDS-PAGE gel and protein bands on the gel were visualized using the Negative Gel Stain MS Kit (Fujifilm Wako Pure Chemical Corporation). Unique bands visualized on the SDS-PAGE gel were cut out, shredded, bleached, and digested with trypsin for MS analysis.

The mutant cDNAs of GALNTL5 tagged with Flag were transfected into GC-2spd(ts) cells or HEK293T cells and cultured overnight. The cell lysates were used for IP. IP was carried out using the DDDDK-tagged Protein Magnetic purification Kit (MBL). The purification products were loaded on an SDS-PAGE gel for western blot analysis.

### MS

Peptide sequences were analyzed with MS (Ultraflex; Bruker Daltoniks). To identify proteins, the raw data were processed using Proteome Discoverer 1.4 (Thermo Fisher Scientific, Inc.) in conjunction with the MASCOT search engine, version 2.6.0 (Matrix Science Inc., Boston, MA, USA).

### Immunofluorescence staining

GALNTL5-GFP/GC-2spd(ts) cells were fixed with MeOH, permeabilized with 0.5% Triton X-100 in PBS, blocked in 1% bovine serum albumin (BSA)/PBS, and stained with antibodies. Alexa Fluor–conjugated species-specific anti-IgG (Thermo Fisher Scientific, Inc.) in 1% BSA/PBS were used as the secondary antibody. Nuclei were stained blue using ProLongGlass Antifade Mountant with NucBlue Stain (Invitrogen). Images were obtained using a fluorescence microscope (Olympus), captured with a digital camera (DP72; Olympus), and formatted with Adobe Photoshop Elements 9.0 software.

## Supplementary information

Supplementary Table 1

Supplementary Table 2

Supplementary Movie 1

Supplementary Movie 2

Supplementary Movie 3

Supplementary Movie 4

Supplymental Figures

Supplementary Movies and Figures legends

Uncropped Western Blots

## Data Availability

All data generated or analyzed during this study are included in this published article and its supplementary information files.

## References

[1] Matzuk MM, Lamb DJ. The biology of infertility: Research advances and clinical challenges. Nat Med. 2008;14:1197–213. 18989307 10.1038/nm.f.1895 PMC3786590

[2] Russell LD, Ettlin RA, Sinha Hikim AP, Clegg ED. Histological and histopathological evaluation of the testis. Clearwater, Florida: Cache River Press; 1990.

[3] Khawar MB, Gao H, Li W. Mechanism of acrosome biogenesis in mammals. Front Cell Dev Biol. 2019. 10.3389/fcell.2019.00195 31620437 10.3389/fcell.2019.00195 PMC6759486

[4] Narimatsu H. Construction of a human glycogene library and comprehensive functional analysis. Glycoconj J. 2004;21:17–24. 15467393 10.1023/B:GLYC.0000043742.99482.01

[5] Rottger S, White J, Wandall HH, Ollvo J-C, Stark A, Bennett EP, et al. Localization of three human polypeptide GalNAc-transferases in HeLa cells suggests initiation of O-linked glycosylation throughout the Golgi apparatus. J Cell Sci. 1998;111:45–60. 9394011 10.1242/jcs.111.1.45

[6] Takasaki N, Tachibana K, Ogasawara S, Matsuzaki H, Hagiuda J, Ishikawa H, et al. A heterozygous mutation of GALNTL5 affects male infertility with impairment of sperm motility. Proc Natl Acad Sci USA. 2014;111:1120–5. 24398516 10.1073/pnas.1310777111 PMC3903224

[7] Hagiuda J, Takasaki N, Oya M, Ishikawa H, Narimatsu H. Mutation of *GALNTL5* gene identified in patients diagnosed with asthenozoospermia. Hum Fertil. 2020;23:226–33. 10.1080/14647273.2018.1562239 30628500

[8] Kasahara Y, Osuka S, Takasaki N, Bayasula, Koya Y, Nakanishi N, et al. Primate-specific POTE-actin gene could play a role in human folliculogenesis by controlling the proliferation of granulosa cells. Cell Death Discov. 2021. 10.1038/s41420-021-00566-1. 10.1038/s41420-021-00566-1 PMC8292509 34285194

[9] Hofmann M-C, Hesst RA, Goldbergt E, Millan JL. Immortalized germ cells undergo meiosis in vitro. Proc Natl Acad Sci USA. 1994;91:5533–7. 8202522 10.1073/pnas.91.12.5533 PMC44030

[10] Ciechanover A, Kwon YT. Protein quality control by molecular chaperones in neurodegeneration. Front Neurosci. 2017. 10.3389/fnins.2017.00185. 10.3389/fnins.2017.00185 PMC5382173 28428740

[11] McCaffrey K, Braakman I. Protein quality control at the endoplasmic reticulum. Essays Biochem. 2016;60:227–35. 27744338 10.1042/EBC20160003

[12] Tannous A, Pisoni GB, Hebert DN, Molinari M. N-linked sugar-regulated protein folding and quality control in the ER. Semin Cell Dev Biol. 2015;41:79–89. 25534658 10.1016/j.semcdb.2014.12.001 PMC4474783

[13] Gupta R, Brunak S. Prediction of glycosylation across the human proteome and the correlation to protein function. Pac Symp Biocomput. 2002;7:310–22. 11928486

[14] Kozlov G, Gehring K. Calnexin cycle – structural features of the ER chaperone system. FEBS J. 2020;287:4322–40. 32285592 10.1111/febs.15330 PMC7687155

[15] Määttänen P, Gehring K, Bergeron JJM, Thomas DY. Protein quality control in the ER: The recognition of misfolded proteins. Semin Cell Dev Biol. 2010;21:500–11. 20347046 10.1016/j.semcdb.2010.03.006

[16] Sifers RN, Brashears-Macatees S, Kiddsll VJ, Muenschii H, Woos SLC. A frameshift mutation results in a truncated alpha 1-Antitrypsin that is retained within the rough endoplasmic reticulum. J Biol Chem. 1988;263:7330–5. 3259232

[17] Koenig PA, Nicholls PK, Schmidt FI, Hagiwara M, Maruyama T, Frydman GH, et al. The E2 ubiquitin-conjugating enzyme UBE2J1 is required for spermiogenesis in mice. J Biol Chem. 2014;289:34490–502. 25320092 10.1074/jbc.M114.604132 PMC4263858

[18] Nagamori I, Yabuta N, Fujii T, Tanaka H, Tomogida K, Nishimune Y, et al. Tisp40, a spermatid specific bZip transciption factor, functions by binding to the unfolded protein response element via the Rip pathway. Genes Cells. 2005;10:575–94. 15938716 10.1111/j.1365-2443.2005.00860.x

[19] Hwang J, Qi L. Quality control in the endoplasmic reticulum: crosstalk between ERAD and UPR pathways. Trends Biochem Sci. 2018;43:593–605. 30056836 10.1016/j.tibs.2018.06.005 PMC6327314

[20] Roth J, Zuber C. Quality control of glycoprotein folding and ERAD: the role of N-glycan handling, EDEM1 and OS-9. Histochem Cell Biol. 2017;147:269–84. 27803995 10.1007/s00418-016-1513-9

[21] Kreft ME, Giandomenico D, Beznoussenko GV, Resnik N, Mironov AA, Jezernik K. Golgi apparatus fragmentation as a mechanism responsible for uniform delivery of uroplakins to the apical plasma membrane of uroepithelial cells. Biol Cell. 2010;102:593–607. 20735355 10.1042/BC20100024

[22] Nakamoto T, Sakai Y. Changes in endoplasmic reticulum during spermiogenesis in the mouse. Cell Tissue Res. 1989;257:279–84. 2776183 10.1007/BF00261831

[23] Clermont Y, Rambourg A. Evolution of the endoplasmic reticulum during rat spermiogenesis. Am J Anat. 1978;151:191–211. 626152 10.1002/aja.1001510204

[24] Hermo L, Clermont Y, Rambourg A. Endoplasmic reticulum‐Golgi apparatus relationships in the rat spermatid. Anat Rec. 1979;193:243–55. 426297 10.1002/ar.1091930206

[25] Li J, Ni M, Lee B, Barron E, Hinton DR, Lee AS. The unfolded protein response regulator GRP78/BiP is required for endoplasmic reticulum integrity and stress-induced autophagy in mammalian cells. Cell Death Differ. 2008;15:1460–71. 18551133 10.1038/cdd.2008.81 PMC2758056

[26] Linda Hendershot RM, Jue-yang Wei FT, Gaut JR, Lawson B, Freiden PJ, Gopal Murti K. In vivo expression of mammalian BiP ATPase mutants causes disruption of the endoplasmic reticulum. Mol Biol Cell. 1995;6:283–96. 7612964 10.1091/mbc.6.3.283 PMC301188

[27] Audouard C, Christians E. Hsp90b1 knockout targeted to male germline: a mouse model for globozoospermia. Fertil Steril. 2011;95:1475–7. 21208614 10.1016/j.fertnstert.2010.12.006

[28] Nagamori I, Yomogida K, Ikawa M, Okabe M, Yabuta N, Nojima H. The testes-specific bZip type transcription factor Tisp40 plays a role in ER stress responses and chromatin packaging during spermiogenesis. Genes Cells. 2006;11:1161–71. 16999736 10.1111/j.1365-2443.2006.01013.x

[29] Nozawa K, Fujihara Y, Devlin DJ, Deras RE, Kent K, Larina IV, et al. The testis-specific E3 ubiquitin ligase RNF133 is required for fecundity in mice. BMC Biol 2022;20. 10.1186/s12915-022-01368-2. 10.1186/s12915-022-01368-2 PMC9277888 35831855

[30] Yao R, Ito C, Natsume Y, Sugitani Y, Yamanaka H, Kuretake S, et al. Lack of acrosome formation in mice lacking a Golgi protein, GOPC. Proc Natl Acad Sci USA. 2002;99:11211–6. 12149515 10.1073/pnas.162027899 PMC123235

[31] Han F, Liu C, Zhang L, Chen M, Zhou Y, Qin Y, et al. Globozoospermia and lack of acrosome formation in GM130-deficient mice. Cell Death Dis. 2017. 10.1038/cddis.2016.414. 10.1038/cddis.2016.414 PMC5386352 28055014

[32] Miyazaki T, Mori M, Yoshida CA, Ito C, Yamatoya K, Moriishi T, et al. Galnt3 deficiency disrupts acrosome formation and leads to oligoasthenoteratozoospermia. Histochem Cell Biol. 2013;139:339–54. 23052838 10.1007/s00418-012-1031-3

[33] Grumati P, Dikic I, Stolz A. ER-phagy at a glance. J Cell Sci. 2018. 10.1242/jcs.217364. 10.1242/jcs.217364 30177506

[34] Lamb CA, Yoshimori T, Tooze SA. The autophagosome: origins unknown, biogenesis complex. Nat Rev Mol Cell Biol. 2013;14:759–74. 24201109 10.1038/nrm3696

[35] Wang M, Zeng L, Su P, Ma L, Zhang M, Zhang YZ. Autophagy: a multifaceted player in the fate of sperm. Hum Reprod Update. 2022;28:200–31. 34967891 10.1093/humupd/dmab043 PMC8889000

[36] Ren C, Li X, Li X, Xie Y, Fu H, Yan Z, et al. RNAi of Grp78 may disturb the fusion of ICR mouse palate cultured in vitro. Hum Exp Toxicol. 2018;37:196–204. 29233047 10.1177/0960327117692132

