# Supplementary material for: GALNTL5, which is restricted to mouse spermatids, impairs endoplasmic reticulum (ER) function through direct interaction with ER chaperone proteins
Source: Cell Death Discov. 2024 Dec 18;10:499. doi: 10.1038/s41420-024-02252-4 (PMC11655647; doi:10.1038/s41420-024-02252-4)
Supplement: Supplementary file 1 — Supplementary Table 1 [file 41420_2024_2252_MOESM1_ESM.pdf]

Supplementary Table 1 List of primers designed to introduce mutations into mouse *Galntl5* and human *GALNTL5* cDNAs

mouse *Galntl5*	sense	anti-sense
N68K	5'-GAGGTTAAGTTTTCAGATCCAGAACTT-3'	5'-TGAAAACTTAACCTCATCATGAGTGAC-3'
N140K	5'-GTGGTGAAGCTCAGTCCGCAGCATCTC-3'	5'-ACTGAGCTTCACCACACTCGACACTGC-3'
N353K	5'-CGTGCAAAGCAGAGTGCCTTGTCAAGG-3'	5'-ACTCTGCTTTGCACGCCTGTGTTGACT-3'
N390K	5'-TGTGGAAAGATTAGTGAGCGCGTGGAA-3'	5'-ACTAATCTTTCCACAGGAAACGTATGT-3'
human *GALNTL5*		
N87K	5'-GATTTTAAGCATACAAACCCAGAACTT-3'	5'-TGTATGCTTAAAATCTGTACCTAACAT-3'
N140K	5'-GTCACGAAGCTCACGCCACACTATTTT-3'	5'-CGTGAGCTTCGTGACACTGGACATGGT-3'

The mutated regions are underlined.
