# Supplementary material for: GALNTL5, which is restricted to mouse spermatids, impairs endoplasmic reticulum (ER) function through direct interaction with ER chaperone proteins
Source: Cell Death Discov. 2024 Dec 18;10:499. doi: 10.1038/s41420-024-02252-4 (PMC11655647; doi:10.1038/s41420-024-02252-4)
Supplement: Supplementary file 2 — Supplementary Table 2 [file 41420_2024_2252_MOESM2_ESM.pdf]

Supplementary Table 2 List of antibodies and appropriate buffers for western blotting

Protein name	maker	Blocking Buffer	Dilution rate
GM130	BD Transduction LaboratoriesTM	CanGet signal	1/250
CREB3L4	Santa Cruz	CanGet signal	1/100
UBE2J1	Santa Cruz	CanGet signal	1/100
IRE1α	Santa Cruz	CanGet signal	1/100
OS9	Abcam	CanGet signal	1/500
GOPC	Abcam	CanGet signal	1/500
Histon H3	Proteintech	CanGet signal	1/2,000
Calnexin	Abcam	5% skim milk in TBST	1/20,000
GRP78 (BiP)	Abcam	5% skim milk in TBST	1/2,000
HSP70	Santa Cruz	5% skim milk in TBST	1/200
HSC70	Abcam	5% skim milk in TBST	1/500
DYKDDDDK	Wako	5% skim milk in TBST	1/1,000
β actin	Wako	5% skim milk in TBST	1/10,000
GAPDH	Wako	5% skim milk in TBST	1/10,000
GFP	Clontech	CanGet signal	1/2,000
DsRed2	Santa Cruz	CanGet signal	1/100
LC3	MBL	5% skim milk in TBST	1/1,000
