# Supplementary material for: GALNTL5, which is restricted to mouse spermatids, impairs endoplasmic reticulum (ER) function through direct interaction with ER chaperone proteins
Source: Cell Death Discov. 2024 Dec 18;10:499. doi: 10.1038/s41420-024-02252-4 (PMC11655647; doi:10.1038/s41420-024-02252-4)
Supplement: Supplementary file 7 — Supplymental Figures [file 41420_2024_2252_MOESM7_ESM.pptx]

## Slide 1
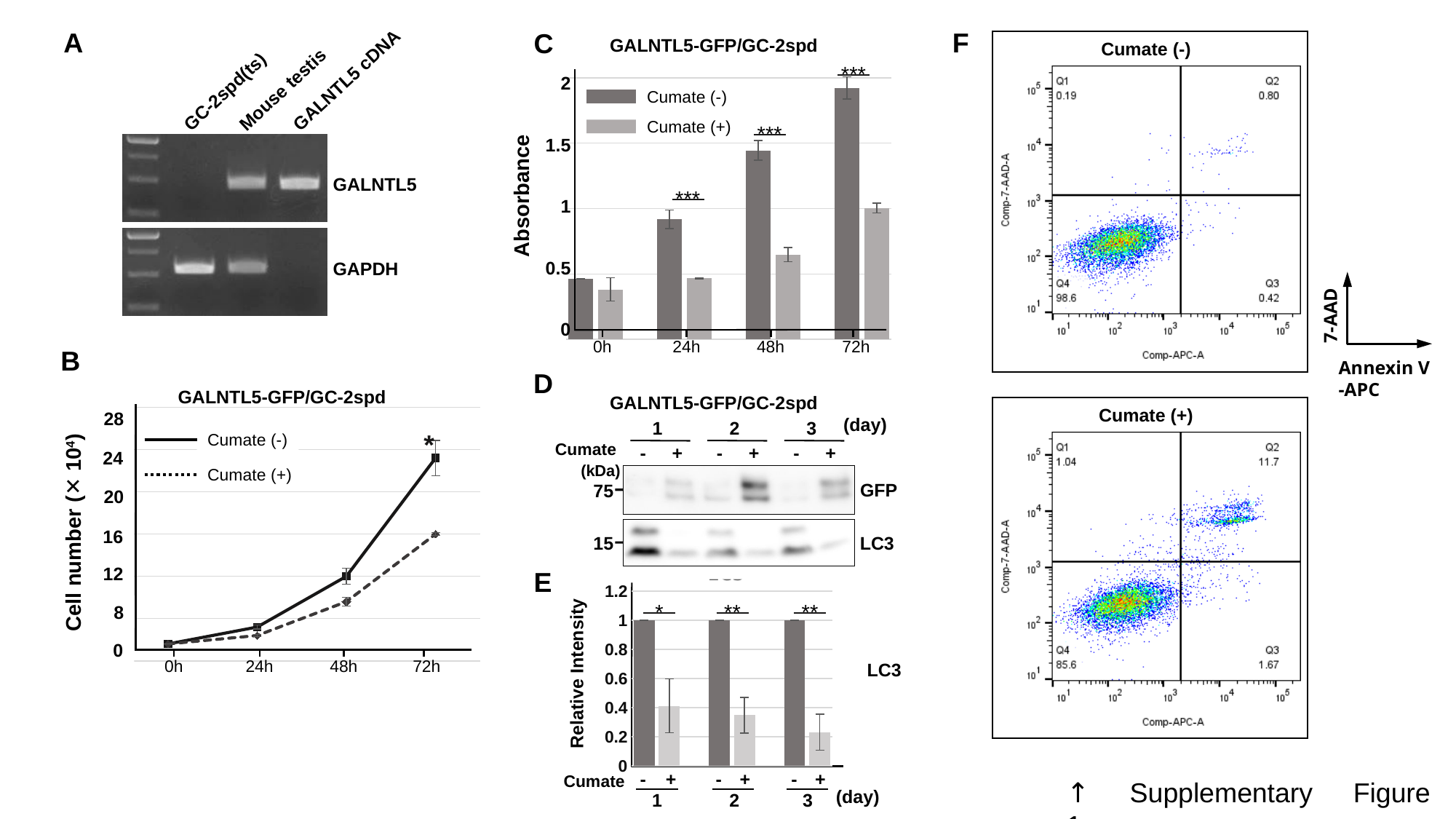

### Chart
| Category | |
|---|---|
| 0 minus | 0.4623333333333333 |
| 0plus | 0.38199999999999995 |
| | None |
| １ON minus | 0.918 |
| １ON plus | 0.4673333333333334 |
| | None |
| ２ON minus | 1.4453333333333334 |
| ２ON plus | 0.6483333333333333 |
| | None |
| ３ON minus | 1.923 |
| 3ON plus | 1.0043333333333333 |
A
F
C
GALNTL5-GFP/GC-2spd
Cumate (-)
***
GALNTL5 cDNA
2
Mouse testis
GC-2spd(ts)
Cumate (-)
Cumate (+)
***
1.5
GALNTL5
Absorbance
***
1
0.5
GAPDH
7-AAD
Annexin V
-APC
0
0h
24h
48h
72h
B
D
GALNTL5-GFP/GC-2spd
GALNTL5-GFP/GC-2spd
Cumate (+)
28
### Chart
| Category | GC23-10 - | GC23-10 + |
|---|---|---|
| 0 | 0.02 | 0.02 |
| 1ON | 0.04 | 0.03 |
| 2ON | 0.1 | 0.07 |
| 3ON | 0.24 | 0.15 |(day)
1
2
3
*
Cumate (-)
Cumate
+
-
+
-
+
-
24
(kDa)
Cumate (+)
75
GFP
20
Cell number (✕ 104)
16
15
LC3
### Chart: LC3
| Category | |
|---|---|
| 1ONm | 1.0 |
| 1ONp | 0.4112386157838781 |
| | None |
| 2ONm | 1.0 |
| 2ONp | 0.3479141296811572 |
| | None |
| 3ONm | 1.0 |
| 3ONp | 0.23186111256909867 |
*
**
**
LC3
Relative Intensity
+
+
+
-
-
-
Cumate
(day)
3
2
1
12
E
8
0
0h
24h
48h
72h
↑　Supplementary　Figure 1

## Slide 2
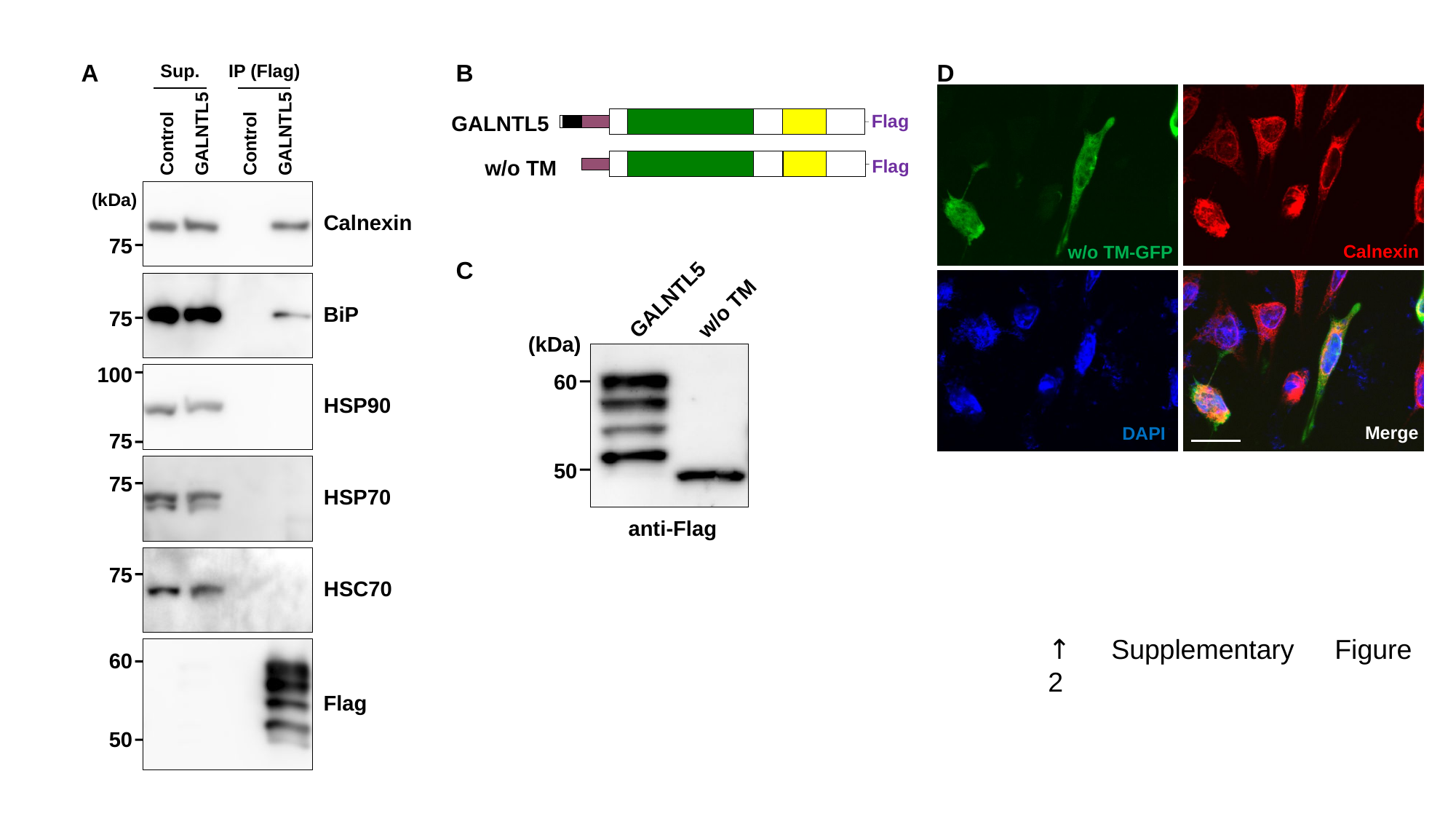

A
B
D
Sup.
IP (Flag)
Flag
GALNTL5
Control
GALNTL5
Control
GALNTL5
w/o TM
Flag
(kDa)
Calnexin
75
Calnexin
w/o TM-GFP
C
GALNTL5
w/o TM
BiP
75
(kDa)
100
60
HSP90
Merge
DAPI
75
50
75
HSP70
anti-Flag
75
HSC70
↑　Supplementary　Figure 2
60
Flag
50

## Slide 3
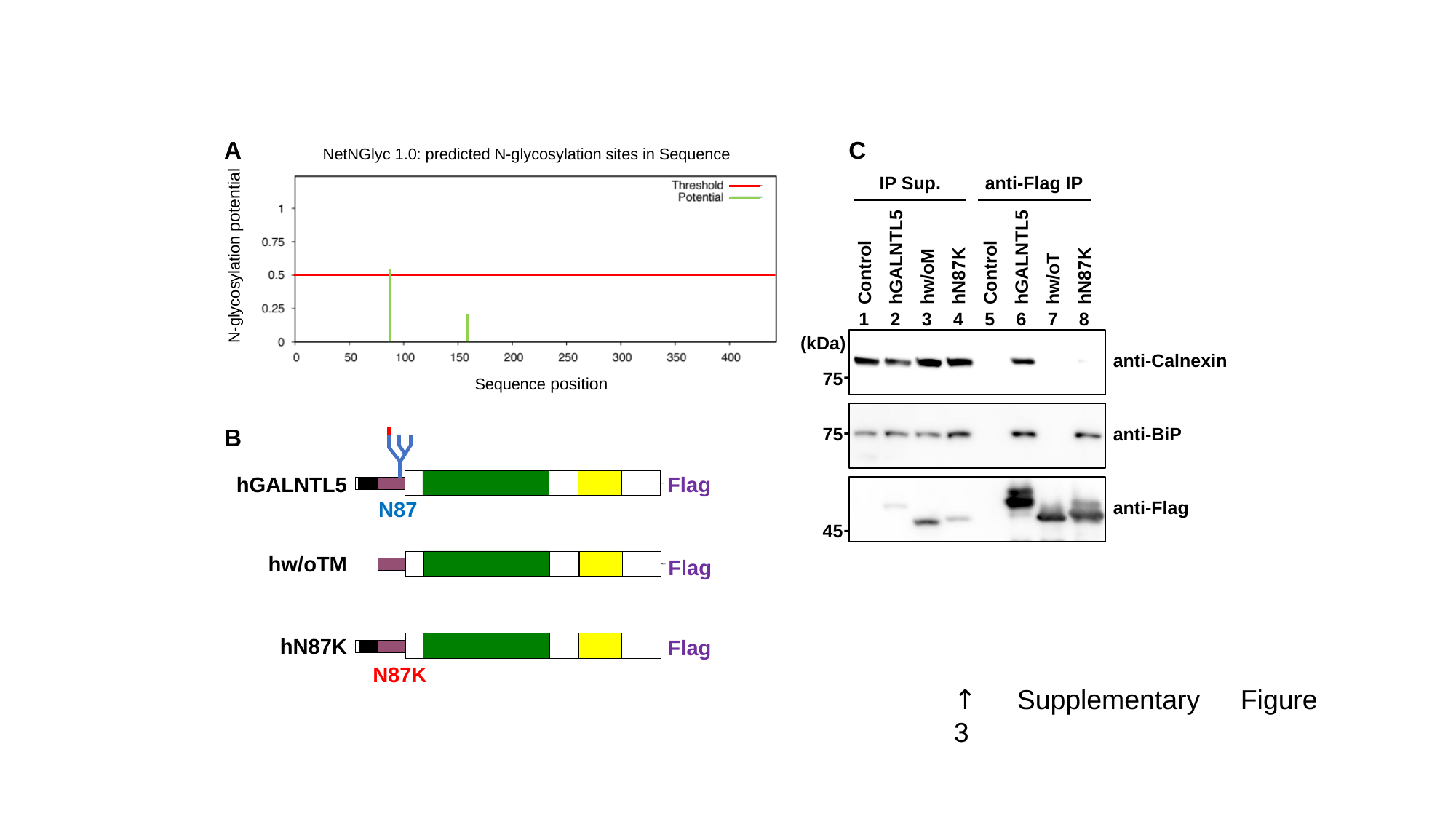

A
C
NetNGlyc 1.0: predicted N-glycosylation sites in Sequence
IP Sup.
anti-Flag IP
hGALNTL5
hGALNTL5
N-glycosylation potential
Control
Control
hw/oM
hw/oT
hN87K
hN87K
1
2
3
4
5
6
7
8
(kDa)
anti-Calnexin
75
Sequence position
75
B
anti-BiP
hGALNTL5
Flag
N87
anti-Flag
45
hw/oTM
Flag
hN87K
Flag
N87K
↑　Supplementary　Figure 3

## Slide 4
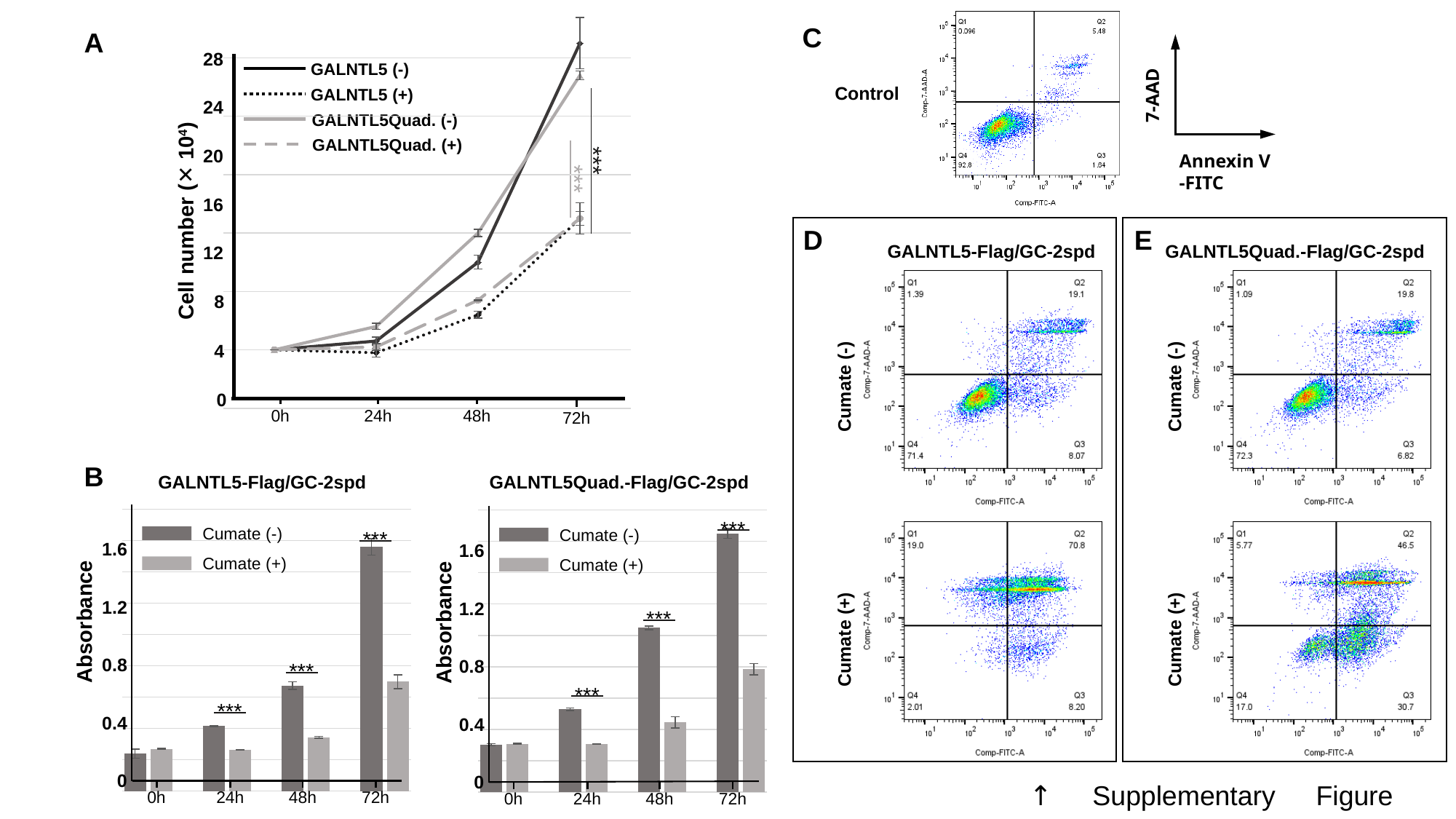

### Chart
| Category | FL6- | FL6+ | Q22- | Q22+ |
|---|---|---|---|---|
| 0 | 0.2 | 0.2 | 0.2 | 0.2 |
| 1ON | 0.23 | 0.19 | 0.28 | 0.21 |
| 2ON | 0.5 | 0.32 | 0.6 | 0.37 |
| 3ON | 1.25 | 0.65 | 1.14 | 0.65 |
28
24
20
16
12
8
4
0
GALNTL5 (-)
GALNTL5 (+)
***
GALNTL5Quad. (-)
GALNTL5Quad. (+)
***
Cell number (✕ 104)
0h
24h
48h
72h
C
A
7-AAD
Annexin V
-FITC
Control
E
D
GALNTL5-Flag/GC-2spd
GALNTL5Quad.-Flag/GC-2spd
Cumate (-)
Cumate (-)
### Chart
| Category |
|---|GALNTL5-Flag/GC-2spd
### Chart
| Category | |
|---|---|
| 0 minus | 0.238 |
| 0plus | 0.268 |
| | None |
| １ON minus | 0.4143333333333333 |
| １ON plus | 0.26233333333333336 |
| | None |
| ２ON minus | 0.6726666666666666 |
| ２ON plus | 0.3433333333333333 |
| | None |
| ３ON minus | 1.5586666666666666 |
| 3ON plus | 0.6973333333333334 |Cumate (-)
***
1.6
Cumate (+)
1.2
Absorbance
0.8
***
***
0.4
0
0h
24h
48h
72h
B
GALNTL5Quad.-Flag/GC-2spd
### Chart
| Category | |
|---|---|
| 0 minus | 0.302 |
| 0plus | 0.308 |
| | None |
| １ON minus | 0.5293333333333333 |
| １ON plus | 0.3053333333333333 |
| | None |
| ２ON minus | 1.0483333333333333 |
| ２ON plus | 0.4463333333333333 |
| | None |
| ３ON minus | 1.6499999999999997 |
| 3ON plus | 0.7839999999999999 |***
Cumate (-)
1.6
Cumate (+)
1.2
***
0.8
***
0.4
0
0h
24h
48h
72h
Absorbance
Cumate (+)
Cumate (+)
↑　Supplementary　Figure 4

## Slide 5
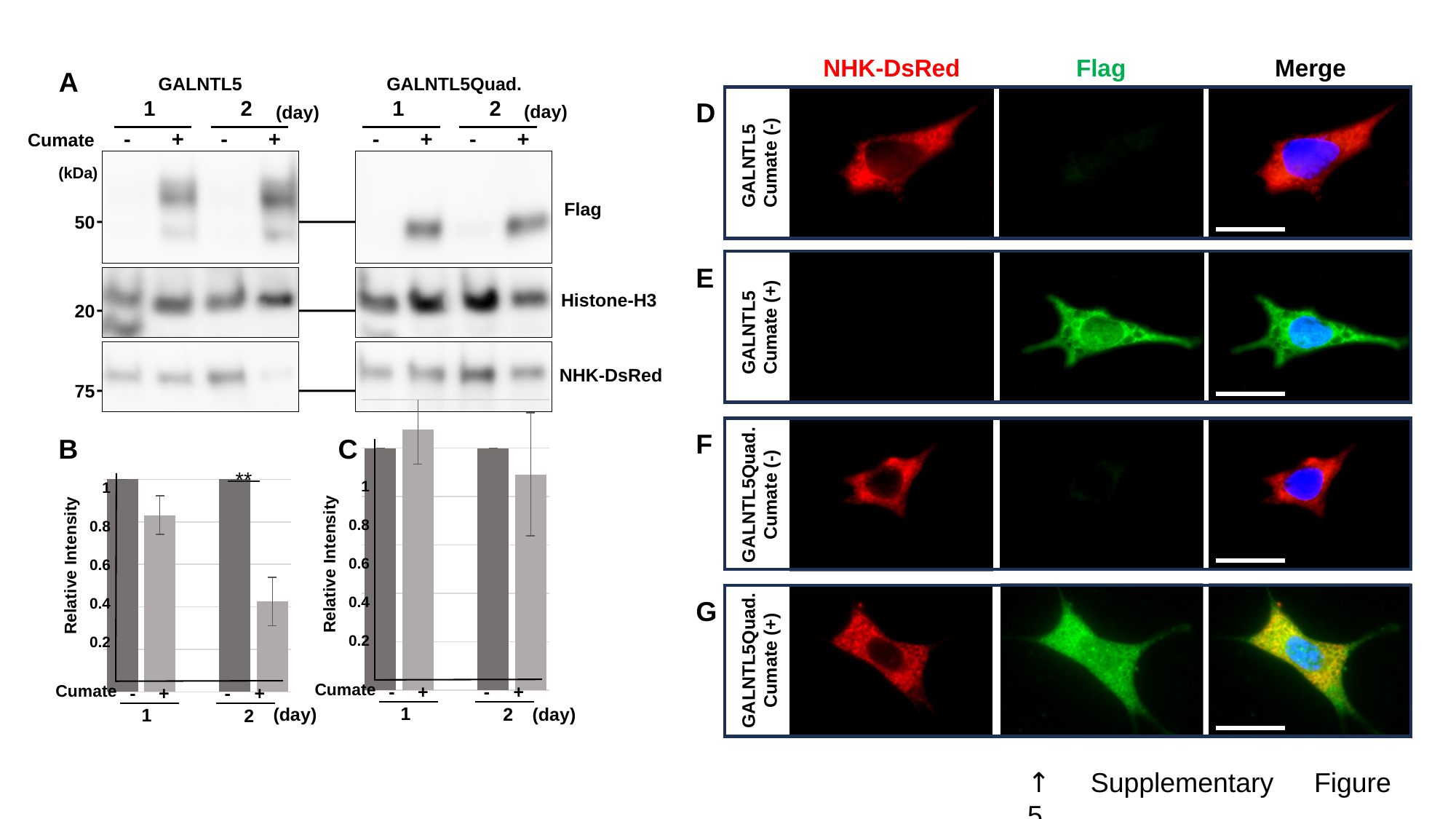

NHK-DsRed
Flag
Merge
GALNTL5
Cumate (-)
GALNTL5
Cumate (+)
GALNTL5Quad.
Cumate (-)
GALNTL5Quad.
Cumate (+)
A
GALNTL5
GALNTL5Quad.
1
-
+
2
-
+
1
-
+
2
-
+
D
(day)
(day)
Cumate
(kDa)
Flag
50
E
Histone-H3
20
NHK-DsRed
75
### Chart
| Category | |
|---|---|
| 1ONm | 1.0 |
| 1ONp | 1.0755027217776871 |
| | None |
| 2ONm | 1.0 |
| 2ONp | 0.8916330424848602 |F
B
### Chart
| Category | |
|---|---|
| 1ONm | 1.0 |
| 1ONp | 0.8311047864145696 |
| | None |
| 2ONm | 1.0 |
| 2ONp | 0.4251515255621168 |**
1
### Chart
| Category |
|---|0.8
Relative Intensity
0.6
0.4
0.2
Cumate
-
+
-
+
(day)
1
2
C
1
0.8
Relative Intensity
0.6
0.4
0.2
Cumate
-
+
-
+
1
(day)
2
### Chart
| Category |
|---|G
↑　Supplementary　Figure 5

## Slide 6
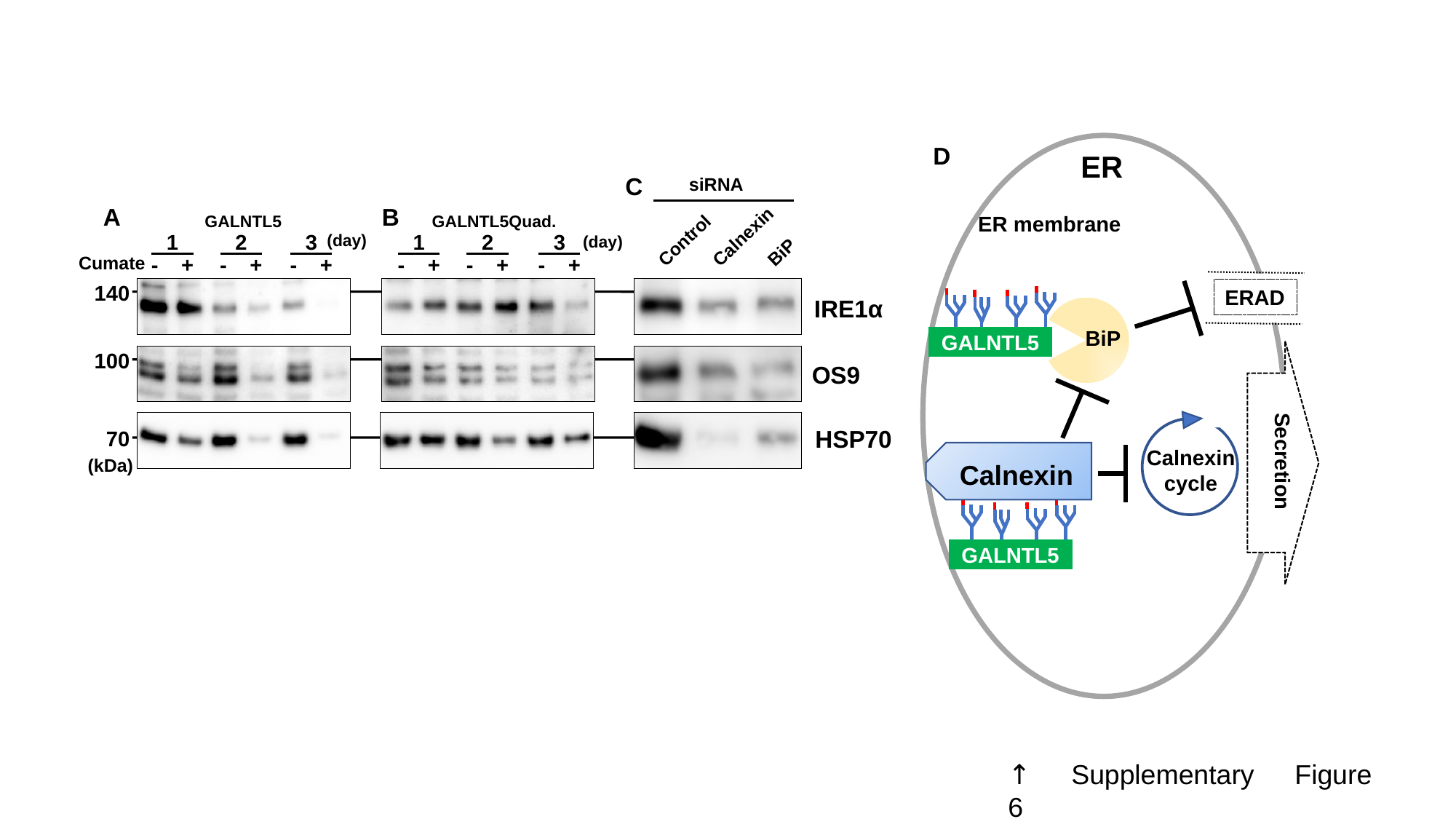

D
ER
C
siRNA
A
B
ER membrane
GALNTL5
GALNTL5Quad.
Calnexin
Control
1
+
-
2
+
-
3
+
-
1
+
-
2
+
-
3
+
-
(day)
(day)
BiP
Cumate
140
ERAD
IRE1α
BiP
GALNTL5
100
Secretion
OS9
HSP70
70
Calnexin cycle
(kDa)
Calnexin
GALNTL5
↑　Supplementary　Figure 6

## Slide 7
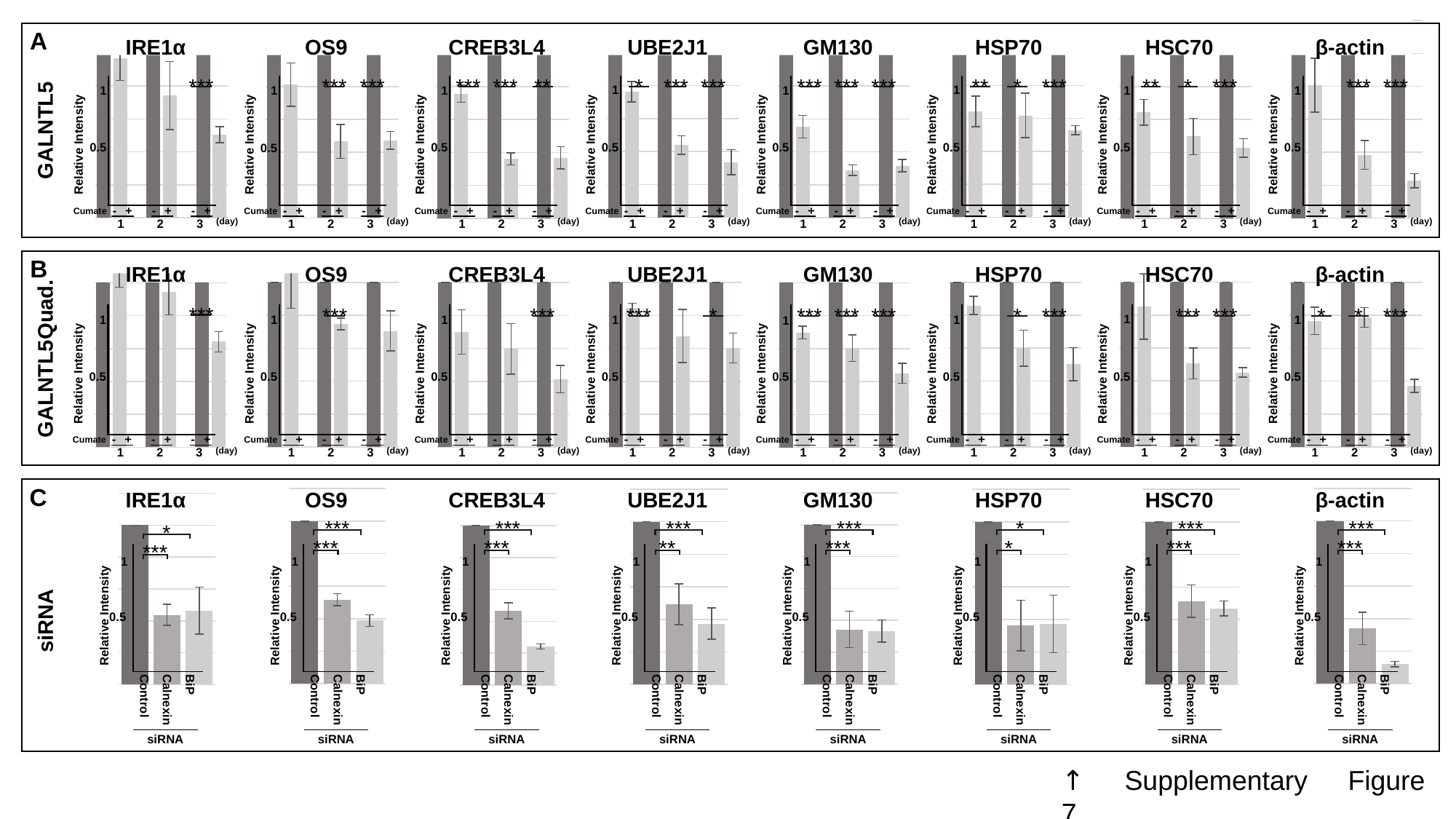

### Chart
| Category | |
|---|---|
| 1ONm | 1.0 |
| 1ONp | 0.6427512492677713 |
| | None |
| 2ONm | 1.0 |
| 2ONp | 0.6183414395838356 |
| | None |
| 3ONm | 1.0 |
| 3ONp | 0.5284871627309327 |**
*
***
1
Relative Intensity
0.5
+
-
1
+
-
2
+
-
3
Cumate
(day)
### Chart
| Category | |
|---|---|
| 1ONm | 1.0 |
| 1ONp | 0.7630459889485358 |
| | None |
| 2ONm | 1.0 |
| 2ONp | 0.439314487493289 |
| | None |
| 3ONm | 1.0 |
| 3ONp | 0.3352805839196276 |*
***
***
1
Relative Intensity
0.5
+
-
1
+
-
2
+
-
3
Cumate
(day)
### Chart
| Category | |
|---|---|
| 1ONm | 1.0 |
| 1ONp | 0.5525883120127305 |
| | None |
| 2ONm | 1.0 |
| 2ONp | 0.2891804859306185 |
| | None |
| 3ONm | 1.0 |
| 3ONp | 0.3160646236684585 |***
***
***
1
Relative Intensity
0.5
+
-
1
+
-
2
+
-
3
Cumate
(day)
### Chart
| Category | |
|---|---|
| 1ONm | 1.0 |
| 1ONp | 0.9700004608697513 |
| | None |
| 2ONm | 1.0 |
| 2ONp | 0.7423792868491663 |
| | None |
| 3ONm | 1.0 |
| 3ONp | 0.5044946448714897 |***
1
Relative Intensity
0.5
+
-
1
+
-
2
+
-
3
Cumate
(day)
### Chart
| Category | |
|---|---|
| 1ONm | 1.0 |
| 1ONp | 0.8078434698759341 |
| | None |
| 2ONm | 1.0 |
| 2ONp | 0.38455821106828486 |
| | None |
| 3ONm | 1.0 |
| 3ONp | 0.22675492843586928 |***
***
1
Relative Intensity
0.5
+
-
1
+
-
2
+
-
3
Cumate
(day)
### Chart
| Category | |
|---|---|
| 1ONm | 1.0 |
| 1ONp | 0.7567460976803639 |
| | None |
| 2ONm | 1.0 |
| 2ONp | 0.3612196581001572 |
| | None |
| 3ONm | 1.0 |
| 3ONp | 0.36726162305830784 |***
***
**
1
Relative Intensity
0.5
+
-
1
+
-
2
+
-
3
Cumate
(day)
### Chart
| Category | |
|---|---|
| 1ONm | 1.0 |
| 1ONp | 0.6443341402332151 |
| | None |
| 2ONm | 1.0 |
| 2ONp | 0.4965944352218303 |
| | None |
| 3ONm | 1.0 |
| 3ONp | 0.4269096749843609 |**
*
***
1
Relative Intensity
0.5
+
-
1
+
-
2
+
-
3
Cumate
(day)
### Chart
| Category | |
|---|---|
| 1ONm | 1.0 |
| 1ONp | 0.8115950654737558 |
| | None |
| 2ONm | 1.0 |
| 2ONp | 0.4649858327561794 |
| | None |
| 3ONm | 1.0 |
| 3ONp | 0.4719610925266468 |A
IRE1α
OS9
CREB3L4
UBE2J1
GM130
HSP70
HSC70
β-actin
***
***
1
Relative Intensity
0.5
+
-
1
+
-
2
+
-
3
Cumate
(day)
GALNTL5
### Chart
| Category | |
|---|---|
| 1ONm | 1.0 |
| 1ONp | 0.8513382326214729 |
| | None |
| 2ONm | 1.0 |
| 2ONp | 0.5052315533004843 |
| | None |
| 3ONm | 1.0 |
| 3ONp | 0.45107975035083 |***
***
1
Relative Intensity
0.5
+
-
1
+
-
2
+
-
3
Cumate
(day)
### Chart
| Category | |
|---|---|
| 1ONm | 1.0 |
| 1ONp | 0.8593992993232176 |
| | None |
| 2ONm | 1.0 |
| 2ONp | 0.5999122674025431 |
| | None |
| 3ONm | 1.0 |
| 3ONp | 0.5028928542191693 |*
***
1
Relative Intensity
0.5
+
-
1
+
-
2
+
-
3
Cumate
(day)
### Chart
| Category | |
|---|---|
| 1ONm | 1.0 |
| 1ONp | 1.0715871571176645 |
| | None |
| 2ONm | 1.0 |
| 2ONp | 0.7465334517296864 |
| | None |
| 3ONm | 1.0 |
| 3ONp | 0.7048638011530152 |***
1
Relative Intensity
0.5
+
-
1
+
-
2
+
-
3
Cumate
(day)
### Chart
| Category | |
|---|---|
| 1ONm | 1.0 |
| 1ONp | 0.7658339867812646 |
| | None |
| 2ONm | 1.0 |
| 2ONp | 0.7870928767972281 |
| | None |
| 3ONm | 1.0 |
| 3ONp | 0.3712643624991645 |*
*
***
1
Relative Intensity
0.5
+
-
1
+
-
2
+
-
3
Cumate
(day)
### Chart
| Category | |
|---|---|
| 1ONm | 1.0 |
| 1ONp | 0.6990923151250105 |
| | None |
| 2ONm | 1.0 |
| 2ONp | 0.5969749302385338 |
| | None |
| 3ONm | 1.0 |
| 3ONp | 0.4132395221859348 |***
1
Relative Intensity
0.5
+
-
1
+
-
2
+
-
3
Cumate
(day)
### Chart
| Category | |
|---|---|
| 1ONm | 1.0 |
| 1ONp | 0.8461594516932713 |
| | None |
| 2ONm | 1.0 |
| 2ONp | 0.6754490306754916 |
| | None |
| 3ONm | 1.0 |
| 3ONp | 0.6026241234185328 |***
*
1
Relative Intensity
0.5
+
-
1
+
-
2
+
-
3
Cumate
(day)
### Chart
| Category | |
|---|---|
| 1ONm | 1.0 |
| 1ONp | 1.0918774156968623 |
| | None |
| 2ONm | 1.0 |
| 2ONp | 0.9426591376038479 |
| | None |
| 3ONm | 1.0 |
| 3ONp | 0.6409507652655186 |
### Chart
| Category | |
|---|---|
| 1ONm | 1.0 |
| 1ONp | 0.6969068721123404 |
| | None |
| 2ONm | 1.0 |
| 2ONp | 0.6027220052839116 |
| | None |
| 3ONm | 1.0 |
| 3ONp | 0.4502599943634487 |***
***
***
1
Relative Intensity
0.5
+
-
1
+
-
2
+
-
3
Cumate
(day)
B
IRE1α
OS9
CREB3L4
UBE2J1
GM130
HSP70
HSC70
β-actin
***
1
GALNTL5Quad.
Relative Intensity
0.5
+
-
+
-
+
-
Cumate
(day)
1
2
3
C
IRE1α
OS9
CREB3L4
UBE2J1
GM130
HSP70
HSC70
β-actin
### Chart
| Category | |
|---|---|
| Cont | 1.0 |
| CANX | 0.33901086567006083 |
| BiP | 0.12087451415306956 |
***
***
1
Relative Intensity
0.5
BiP
Control
Calnexin
siRNA
### Chart
| Category | |
|---|---|
| Cont | 1.0 |
| CANX | 0.5166470928039494 |
| BiP | 0.38836965206410357 |
***
***
1
Relative Intensity
0.5
BiP
Control
Calnexin
siRNA
### Chart
| Category | |
|---|---|
| Cont | 1.0 |
| CANX | 0.4928685222865468 |
| BiP | 0.37406935671251756 |
***
**
1
Relative Intensity
0.5
BiP
Control
Calnexin
siRNA
### Chart
| Category | |
|---|---|
| Cont | 1.0 |
| CANX | 0.3614592680782361 |
| BiP | 0.37259281119375504 |
*
*
1
Relative Intensity
0.5
BiP
Control
Calnexin
siRNA
### Chart
| Category | |
|---|---|
| Cont | 1.0 |
| CANX | 0.5133270334450771 |
| BiP | 0.4673357263264126 |
***
***
1
Relative Intensity
0.5
BiP
Control
Calnexin
siRNA
### Chart
| Category | |
|---|---|
| Cont | 1.0 |
| CANX | 0.3436575654878908 |
| BiP | 0.33382244158721536 |
***
***
1
Relative Intensity
0.5
BiP
Control
Calnexin
siRNA
### Chart
| Category | |
|---|---|
| Cont | 1.0 |
| CANX | 0.4370998121016199 |
| BiP | 0.46406047609846773 |
*
***
1
Relative Intensity
0.5
BiP
Control
Calnexin
siRNA
### Chart
| Category | |
|---|---|
| Cont | 1.0 |
| CANX | 0.4652874948485292 |
| BiP | 0.24164501105681468 |
***
***
1
Relative Intensity
0.5
BiP
Control
Calnexin
siRNA
siRNA
↑　Supplementary　Figure 7
