# Supplementary material for: GALNTL5, which is restricted to mouse spermatids, impairs endoplasmic reticulum (ER) function through direct interaction with ER chaperone proteins
Source: Cell Death Discov. 2024 Dec 18;10:499. doi: 10.1038/s41420-024-02252-4 (PMC11655647; doi:10.1038/s41420-024-02252-4)
Supplement: Supplementary file 8 — Supplementary Movies and Figures legends [file 41420_2024_2252_MOESM8_ESM.docx]

**Supplementary Movies and Figures legends**

**Supplementary Movie 1**

**Time-lapse microscopy of GALNTL5-GFP/GC-2spd cells in the absence of cumate solution.**

Time-lapse video consisting of 254 frames taken at 17-minute intervals over a duration of about 72 hours in only the phase-contrast channel. A time stamp (h:min) is shown.

**Supplementary Movie 2**

**Time-lapse microscopy of GALNTL5-GFP/GC-2spd cells with continuous expression of GALNTL5-GFP induced by cumate.**

Time-lapse video consisting of 254 frames taken at 17-minute intervals over a duration of about 72 hours in only the phase-contrast channel. A time stamp (h:min) is shown.

**Supplementary Movie 3**

**Time-lapse microscopy of GALNTL5-GFP/GC-2spd cells induced by GALNTL5-GFP in the presence of cumate solution**

Time-lapse video beginning 24 hours after cumate reagent was added. The video consists of 258 frames taken at 10-minutes intervals over a duration of about 43 hours. Two channels, phase contrast and GFP (fluorescence wavelength; 525 ± 25), were collected and merged. A time stamp (h:min) is shown.

**Supplementary Movie 4**

**Time-lapse microscopy of normal GC-2spd(ts) cells in the presence of cumate solution.**

Time-lapse video beginning 24 hours after cumate reagent was added. The video consists of 190 frames taken at 15-minute intervals over a duration of about 48 hours in only the phase-contrast channel. A time stamp (h:min) is shown.

**Supplementary Figure 1**

**Detection of GALNTL5 transcripts and effects of drug-induced expression of GALNTL5 protein in GC-2spd(ts) cells**

**A** Expression of the *Galntl5* gene detected by RT-PCR with primers specific for *Galntl5* mRNA. Total RNA from the GC-2spd(ts) cell line or mouse testis was added to a reverse transcription reaction. GALNTL5 and GAPDH RT-PCR products were amplified from the expected respective cDNAs. *Galntl5* cDNA was used as a positive control for primers specific for *Galntl5* mRNA. **B** Analysis of the number of GALNTL5-GFP/GC-2spd cells counted every 24 h, with or without cumate treatment. The averages (± SEM) of three experimental series were plotted. Two-tailed Student’s t-test with or without cumate treatment after 72 h. *p<0.05. **C** Bromodeoxyuridine (BrdU) proliferation assay of GALNTL5-GFP/GC-2spd cells with or without cumate treatment. The incorporation of BrdU was measured using an ELISA system. Data are presented as means ± SD. Three independent experiments showed similar results. ***p<0.001. **D** Relative levels of target protein in continuous culture of GALNTL5-GFP/GC-2spd cells without or with cumate solution for 3 days. GALNTL5-GFP expression with chemical induction was detected persistently for 3 days. LC3 expression decreased with continuous GALNTL5 expression. **E** Quantification of western blot image with anti-LC3 antibody. Band intensities were analyzed and compared using ImageJ software. Experiments were independently performed in at least three repeats. The error bars are presented as mean ± SEM. *p<0.05, **p<0.01. **E** Alteration of apoptosis in GALNTL5-GFP/GC-2spd cells treated with or without cumate for 72 h, stained with fluorochrome-labeled Annexin V, and analyzed using flow cytometry.

**Supplementary Figure 2**

**The interaction of GALNTL5 with chaperone proteins and the features of mutant GALNTL5, excluding the transmembrane domain.**

**A** Co-IP with anti-Flag antibody using normal GC-2spd(ts) cells transiently transfected with Flag-tagged mouse GALNTL5 cDNA. Treatment with anti-calnexin and anti-BiP antibodies confirmed that ER-resident calnexin and BiP chaperone proteins interact with GALNTL5. Western blotting with antibodies to other chaperon proteins (HSP90, HSP70, and HSC70) showed no direct interactions. Four immunoprecipitates were identified by western blotting with anti-Flag antibody. **B** Schematic comparison of mouse GALNTL5 with or without the transmembrane domain. Normal GALNTL5 possesses a transmembrane domain (back), a stem region (purple), a catalytic unit consisting of a GT1 motif (green) and a Gal/GalNAc-T motif (yellow), and a Flag tag at the C-terminus. “w/o TM” indicates normal GALNTL5 without the transmembrane domain. **C** Transient protein products from normal GALNTL5 cDNA or GALNTL5 cDNA (w/o TM) are lacking the transmembrane in the GC-2spds(ts). Anti-Flag antibody detected four protein bands from normal GALNTL5 cDNA and only one band from w/o TM. **D** Fluorescence image of GALNTL5 (w/o TM-GFP) removed the transmembrane and tagged with GFP at the C-terminal domain in GC-2spds(ts) cells with anti-calnexin antibody, or DAPI nuclear staining. In the merged image, the signal of w/o TM-GFP is distributed throughout the cytoplasm and is not localized in ER stained with anti-calnexin antibody. White scale bars, 20 µm.

**Supplementary Figure 3**

**Direct interactions of human GALNTL5 with calnexin and BiP, the former through *N*-glycosylated modification.**

**A** A search program for *N*-glycosylation sites (https://services.healthtech.dtu.dk/services/NetNGlyc-1.0/) predicted that two asparagine residues (87 and 159) were *N*-glycosylation sites in human GALNTL5 protein. **B** Schematic comparison of human GALNTL5 based on IP experiments. Human GALNTL5 also contains a transmembrane domain (back), a stem region (purple), a catalytic unit consisting of a GT1 motif (green) and a Gal/GalNAc-T motif (yellow), and a Flag tag at the C-terminus. N87 indicates asparagine residues that are N-glycosylation sites in human GALNTL5. hw/o TM consists of human GALNTL5 without the transmembrane domain. In hN87K, asparagine was substituted for lysine at the 87th amino acid. **C** IP with anti-Flag antibody followed by western blotting with anti-Flag antibody. Co-IP with anti-Flag antibody followed by western blotting with anti-calnexin and anti-BiP antibodies.

**Supplementary Figure 4**

**Effects of chemically induced expression of normal GALNTL5 or quadruple mutant GALNTL5 protein in GC-2spd(ts) cells**

**A** Analysis of the number of GALNTL5-Flag/GC-2spd or GALNTL5Quad-Flag/GC-2spd cells treated with or without cumate induction. Cells were counted every 24 h. The averages (± SEM) of three experimental series were plotted. Two-tailed Student’s t-test of each cell line with or without cumate treatment after 72 h. ***p<0.001. **B** BrdU proliferation assay of GALNTL5-Flag/GC-2spd or GALNTL5Quad-Flag/GC-2spd cells with or without cumate treatment. BrdU incorporation was measured using an ELISA system. Data are presented as means ± SD. Three independent experiments showed similar results. ***p<0.001 **C-E** Differences in apoptosis of GALNTL5-Flag/GC-2spd (D) or GALNTL5Quad-Flag/GC-2spd (E) cells with or without drug induction. Cells were stained with fluorochrome-conjugated Annexin V and analyzed using flow cytometry. The control refers to the rate of apoptosis in GC-2spd cells cultured for 72 h (C).

**Supplementary Figure 5**

**Calnexin function is impaired with normal GALNTL5 but not with quadruple mutant GALNTL5**

**A** Transient expression of NHK tagged with DsRed2 in GALNTL5-Flag/GC-2spd or GALNTL5Quad-Flag/GC-2spd cells. NHK-DsRed signals decreased with persistent, 2-day GALNTL5 expression in GALNTL5-Flag/GC-2spd cells, but not with mutant GALNTL5 expression in GALNTL5Quad-Flag/GC-2spd cells. Histone-H3 was used as the control. **B** Quantification of band intensity of NHK-DsRed signals was statistically analyzed in GALNTL5-Flag/GC-2spd cells (mean ± SEM. n = 3. Two-tailed Student’s t-test. **p<0.01). **C** Quantification of band intensity of NHK-DsRed signals was statistically analyzed in GALNTL5Quad-Flag/GC-2spd cells (mean ± SEM. n = 3). **D, E** Fluorescence images of GALNTL5-Flag/GC-2spd cells transiently expressing NHK tagged with DsRed2. Without the induction of GALNTL5-Flag expression, the NHK-DsRed signals localize in the ER (D). When the expression of GALNTL5-Flag was induced with cumate solution, NHK-DsRed signals disappeared from the ER harboring GALNTL5-Flag 2 days later, and the ER became enlarged with a rough mesh structure (E). **F, G** Fluorescence images of GALNTL5quad-Flag/GC-2spd cells transiently expressing NHK tagged with DsRed2. Without the induction of GALNTL5quad-Flag expression, the NHK-DsRed signals localize in the ER (F). When the expression of GALNTL5quad-Flag was induced with cumate solution, NHK-DsRed signals still colocalized with GALNTL5quad-Flag signals in the ER 2 days later in a fine mesh pattern (G). Nuclei are stained with DAPI in the merged image. White scale bars in the merged image, 20 µm.

**Supplementary Figure 6**

**Influences of GALNTL5, a quadruplet GALNTL5 mutant, and siRNA knockdown of proteins in GC-2spd(ts) cells.**

**A** Continuous culture of GALNTL5-Flag/GC-2spd cells with or without cumate solution for 3 days. With cumate induction (lanes indicated by +), the levels of two ER proteins (IRE1α and OS9) and a cytoplasmic protein (HSP70) were hardly detected by western blotting after 3 days. **B** Continuous culture of GALNTL5Quad-Flag/GC-2spd cells with or without cumate solution for 3 days. Compared with GALNTL5-Flag/GC-2spd, there are slight declines in the levels of the three proteins (IRE1α, OS9, and HSP70) after cumate induction for 3 days. **C** siRNA knockdown of calnexin or BiP in GC-2spd(ts) cells reduced the amounts of the three proteins (IRE1α, OS9, and HSP70). **D** Model of GALNTL5 localized in the ER. GALNTL5 is tethered to the ER membrane through its transmembrane domain. The *N*-glycan on GALNTL5 recognizes and binds to calnexin. This interaction impairs the calnexin cycle and simultaneously attenuates BiP function. In parallel, the association between BiP and GALNTL5 also interferes with the ERAD system.

**Supplementary Figure 7**

**Histograms of western blot signal intensities after cell culture with antibodies.**

**A** Quantification of signal intensities detected with anti-IRE1α, anti-OS9, anti-CREB3L4, anti-UBE2J1, anti-GM130, anti-HSP-70, anti-HSC70, and anti-β-actin antibodies in continuous culture with GALNTL5-Flag/GC-2spd cells with or without cumate solution for 3 days. **B** Quantification of signal intensities detected with anti-IRE1α, anti-OS9, anti-CREB3L4, anti-UBE2J1, anti-GM130, anti-HSP-70, anti-HSC70, and anti-β-actin antibodies in continuous culture with GALNTL5Quad-Flag/GC-2spd cells with or without cumate solution for 3 days. **C** Quantification of signal intensities detected with anti-IRE1α, anti-OS9, anti-CREB3L4, anti-UBE2J1, anti-GM130, anti-HSP-70, anti-HSC70, and anti-β-actin antibodies in three categories of GC-2spd(ts) cells: 72-h transfection with control, calnexin or BiP siRNA. All values are means ± SEM (error bars, n=3). Two-tailed Student’s t-test *p<0.05, **p<0.01, ***p<0.001.
