# Supplementary material for: GALNTL5, which is restricted to mouse spermatids, impairs endoplasmic reticulum (ER) function through direct interaction with ER chaperone proteins
Source: Cell Death Discov. 2024 Dec 18;10:499. doi: 10.1038/s41420-024-02252-4 (PMC11655647; doi:10.1038/s41420-024-02252-4)
Supplement: Supplementary file 9 — Uncropped Western Blots [file 41420_2024_2252_MOESM9_ESM.pdf]

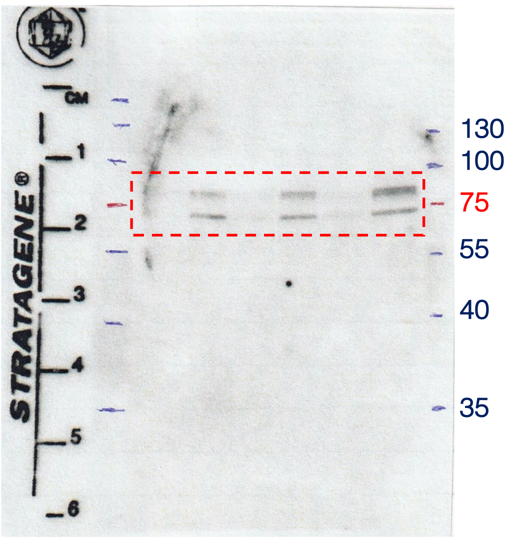

Figure 1A
GFP

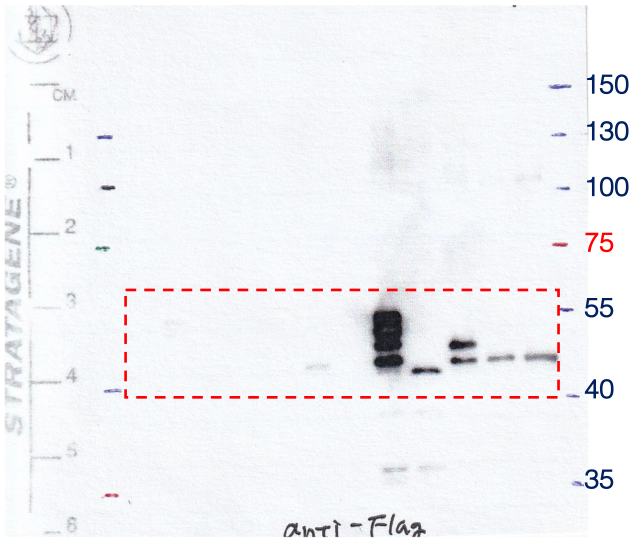

Figure 3
Flag

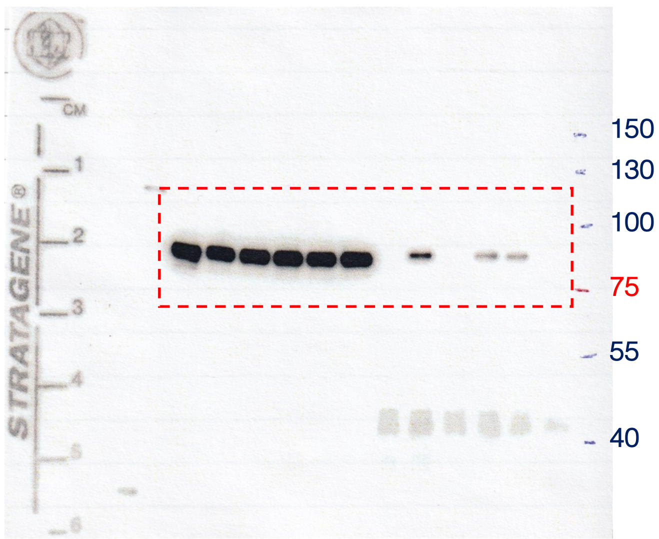

Figure 3
Calnexin

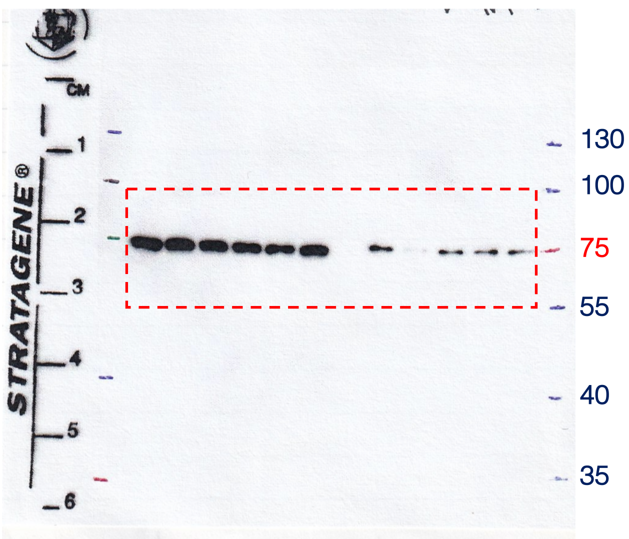

Figure 3
BiP

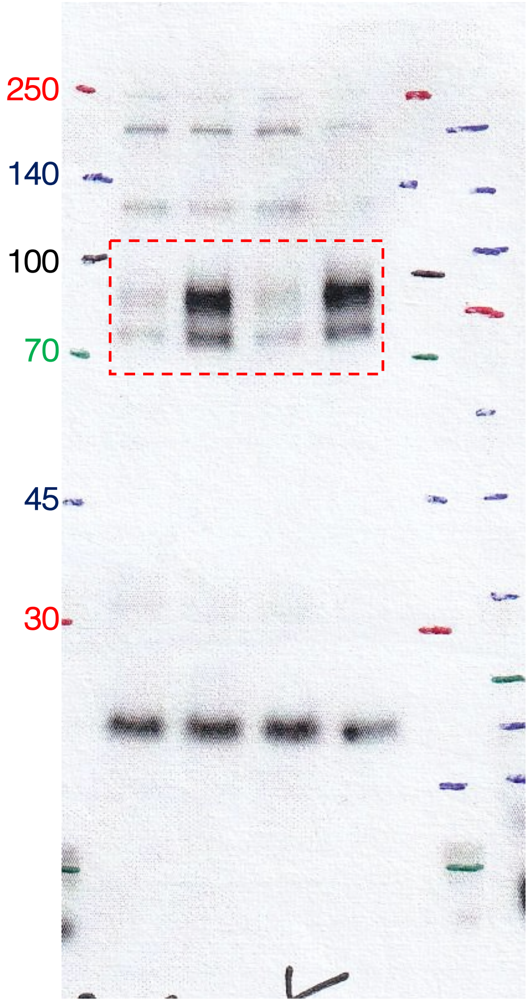

Figure 4C
GFP

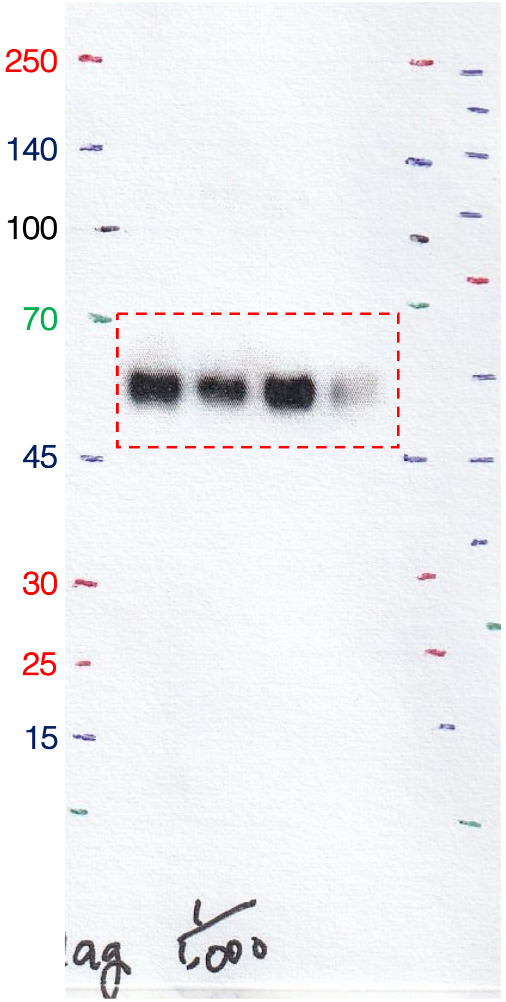

Figure 4C
NHK-Flag

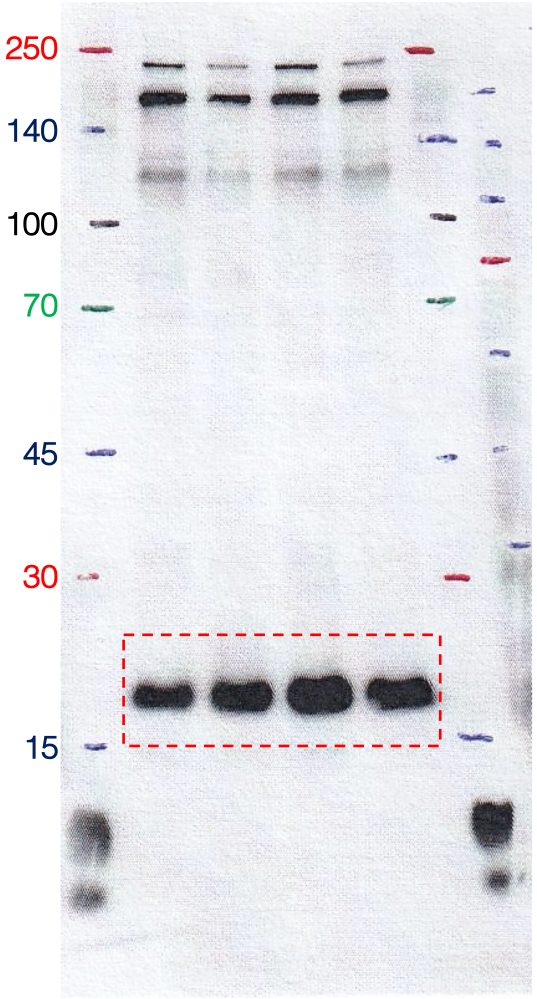

Figure 4C
Histone-H3

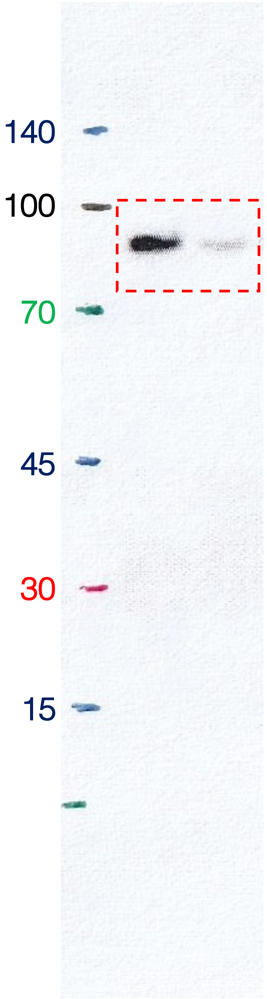

Figure 4E
Calnexin

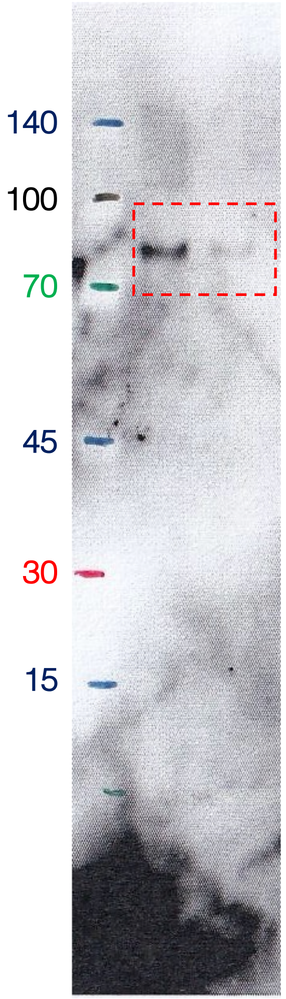

Figure 4E
NHK-DsRed

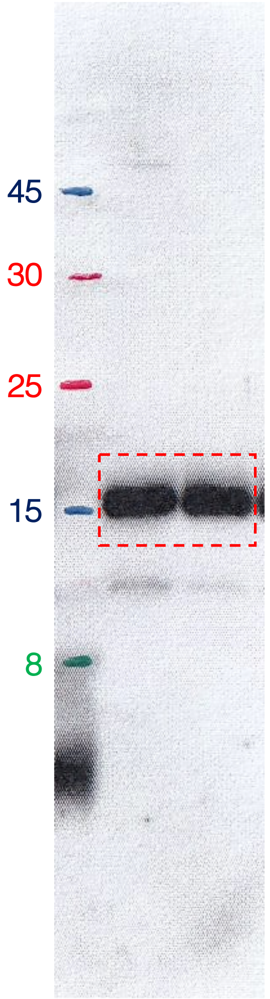

Figure 4E
Hsitone-H3

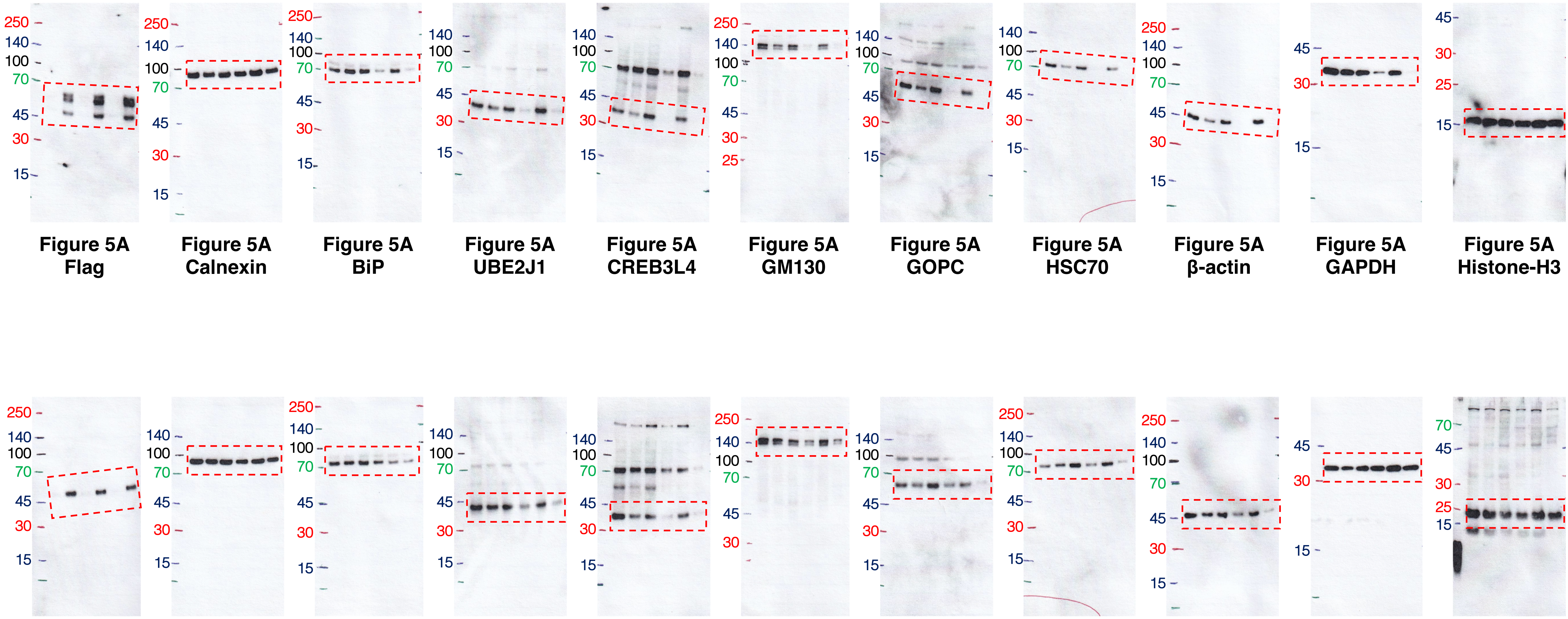

Figure 5A Flag | Figure 5A Calnexin | Figure 5A BiP | Figure 5A UBE2J1 | Figure 5A CREB3L4 | Figure 5A GM130 | Figure 5A GOPC | Figure 5A HSC70 | Figure 5A β-actin | Figure 5A GAPDH | Figure 5A Histone-H3

Figure 5B Flag | Figure 5B Calnexin | Figure 5B BiP | Figure 5B UBE2J1 | Figure 5B CREB3L4 | Figure 5B GM130 | Figure 5B GOPC | Figure 5B HSC70 | Figure 5B β-actin | Figure 5B GAPDH | Figure 5B Histone-H3

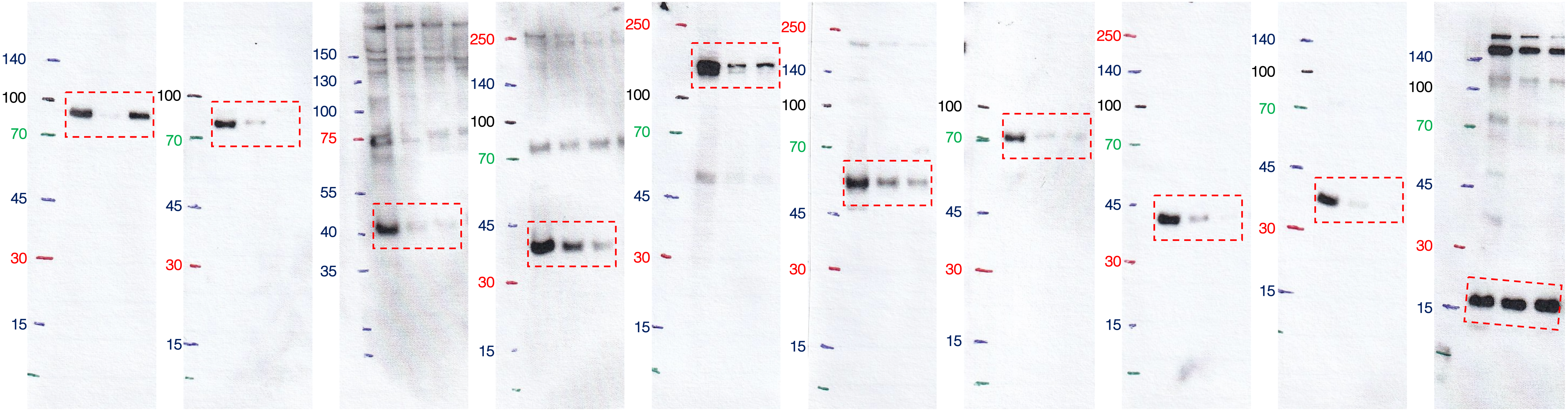

Figure 5G Calnexin | **Figure 5G BiP** | **Figure 5G UBE2J1** | **Figure 5G CREB3L4** | **Figure 5G GM130** | **Figure 5G GOPC** | **Figure 5G HSC70** | **Figure 5G β-actin** | **Figure 5G GAPDH** | **Figure 5G Histone-H3**

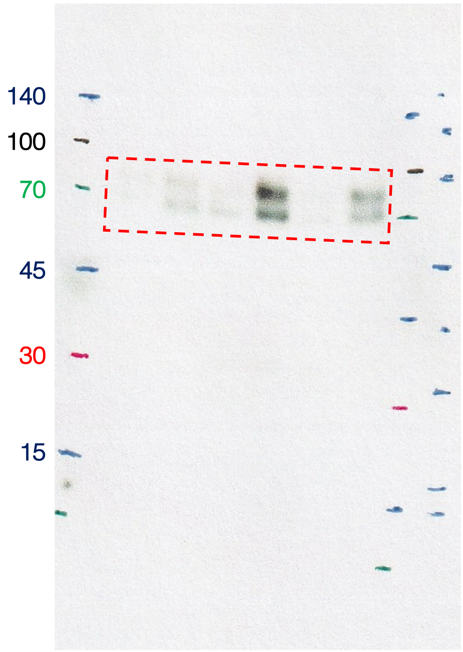

Supplementary Figure 1D GFP

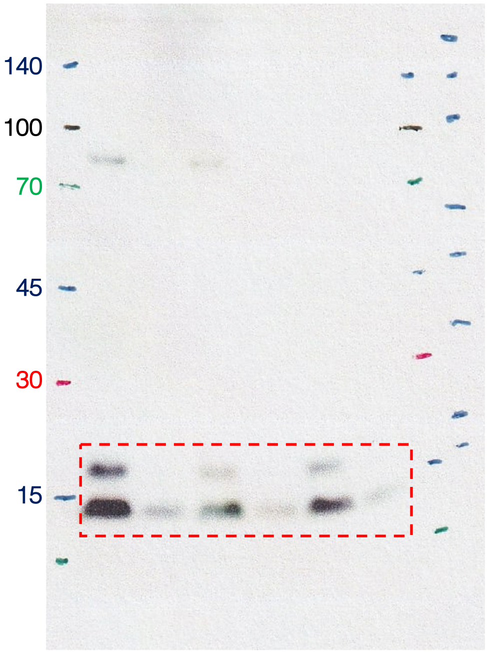

Supplementary Figure 1D LC3

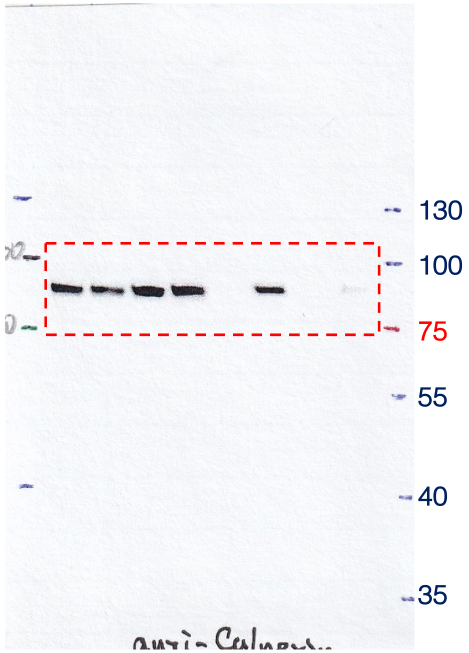

Supplementary Figure 3C Calnexin

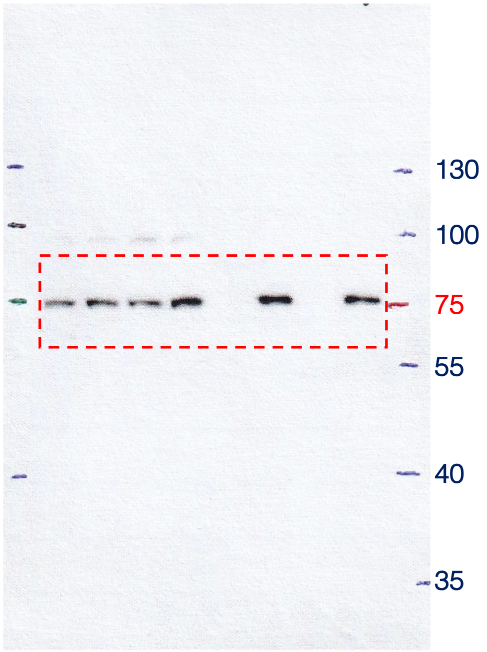

Supplementary Figure 3C BiP

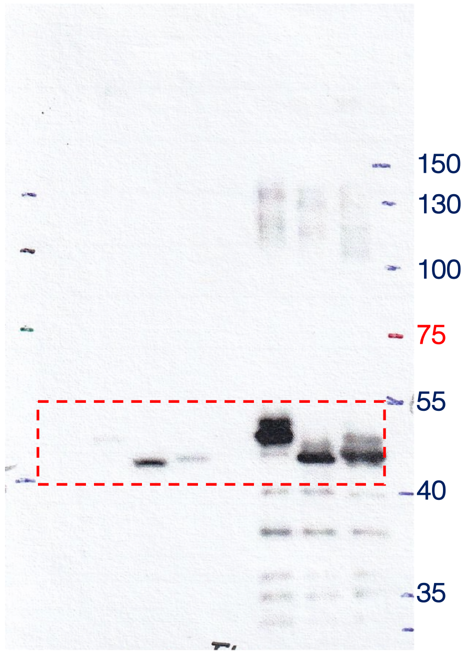

Supplementary Figure 3C Flag

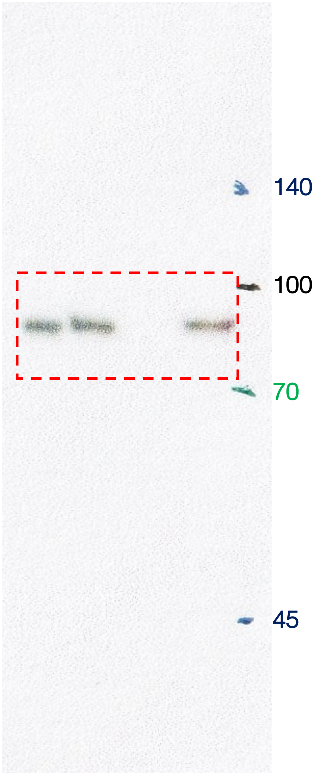

Supplementary Figure 2A Calnexin

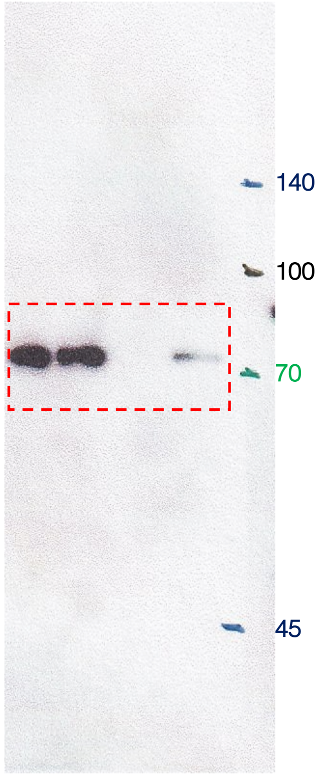

Supplementary Figure 2A BiP

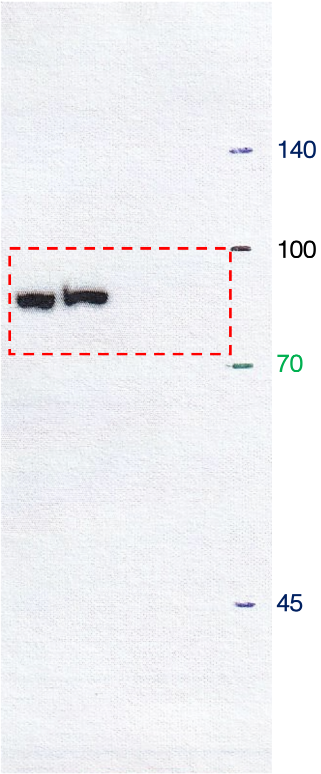

Supplementary Figure 2A HSP90

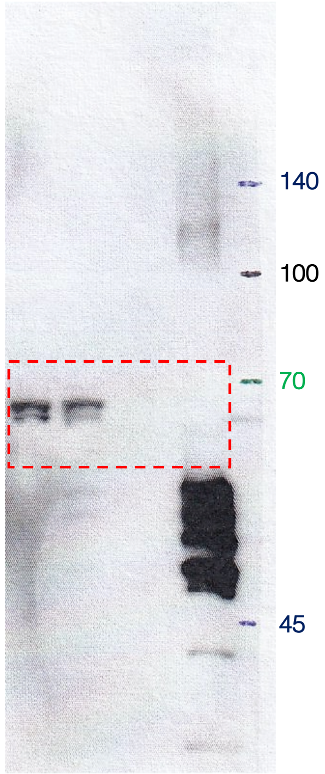

Supplementary Figure 2A HSP70

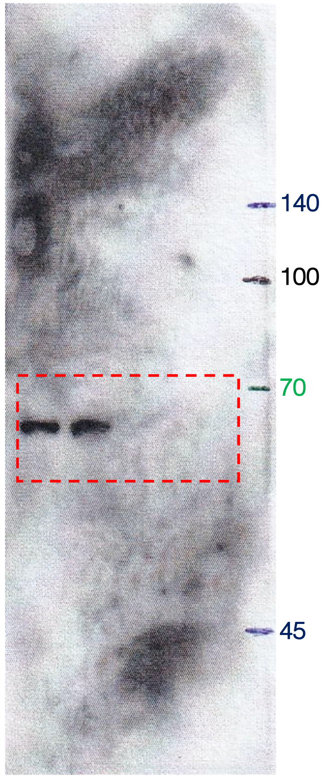

Supplementary Figure 2A HSC70

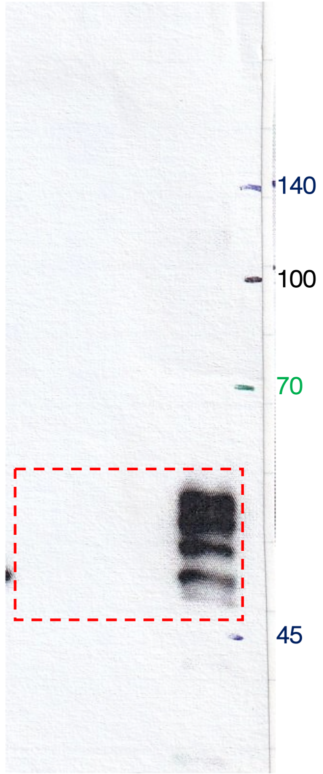

Supplementary Figure 2A Flag

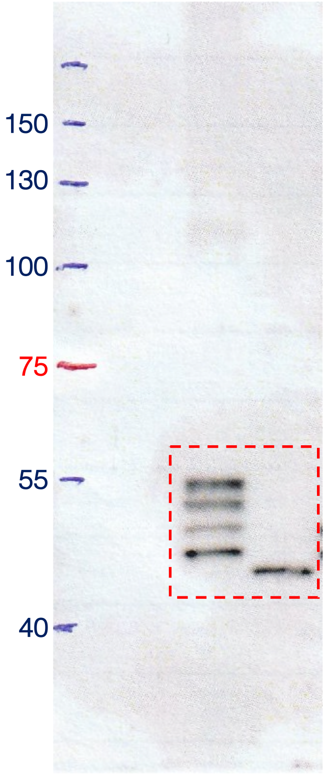

Supplementary Figure 2A anti-Flag

GALNTL5 | GALNTL5Quad.

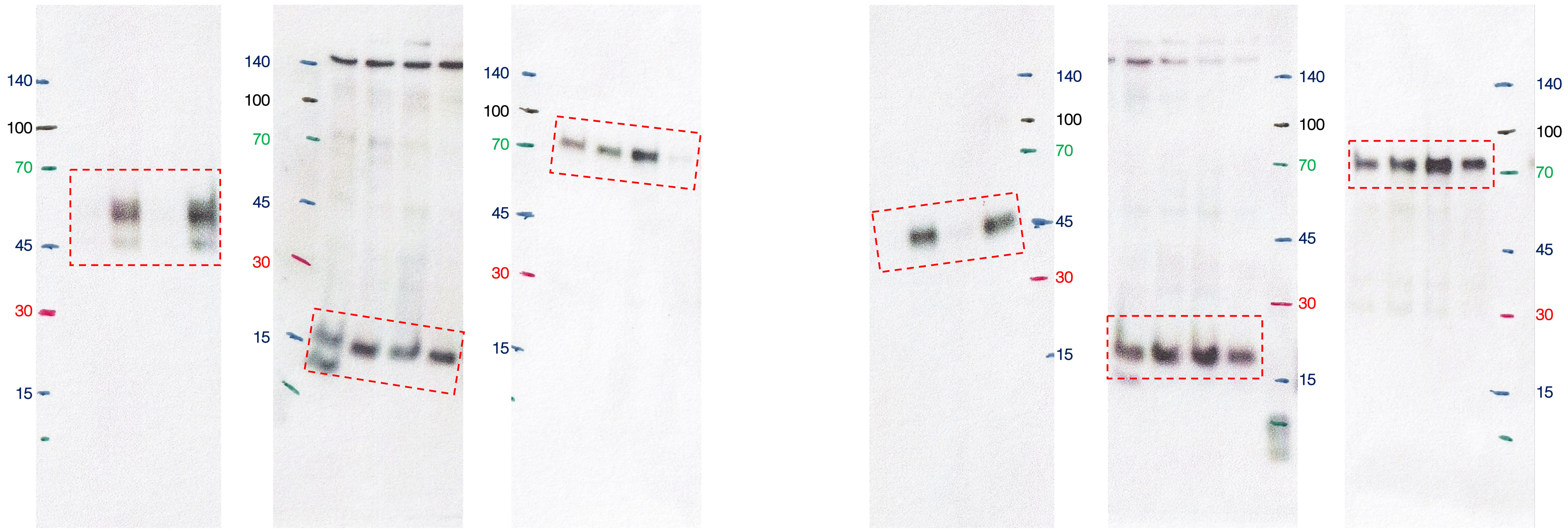

Supplementary Figure 5A Flag

Supplementary Figure 5A Histone-H3

Supplementary Figure 5A NHK-DsRed

Supplementary Figure 5A Flag

Supplementary Figure 5A Histone-H3

Supplementary Figure 5A NHK-DsRed

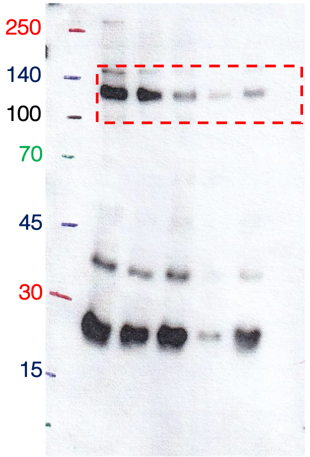

Supplementary Figure 6A IRE1α

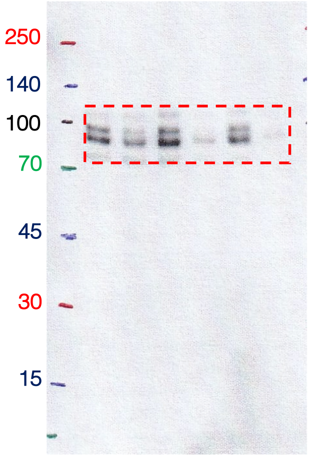

Supplementary Figure 6A OS9

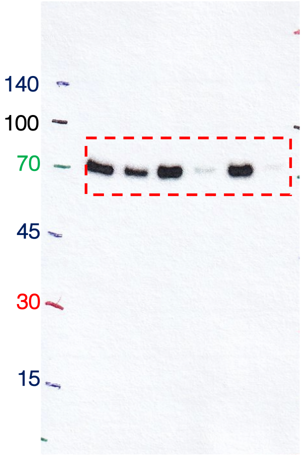

Supplementary Figure 6A HSP70

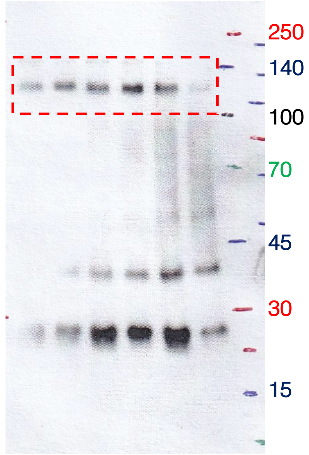

Supplementary Figure 6B IRE1α

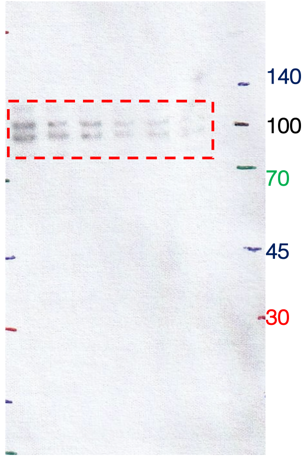

Supplementary Figure 6B OS9

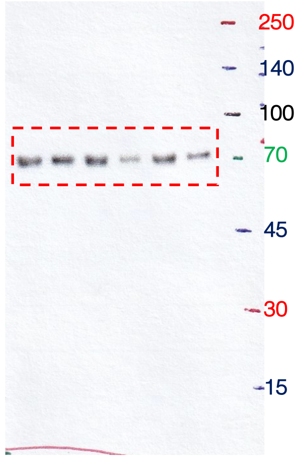

Supplementary Figure 6B HSP70

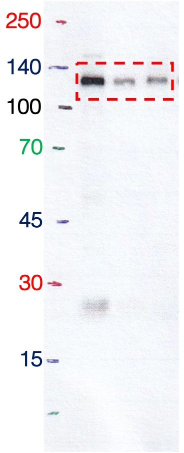

Supplementary Figure 6C IRE1α

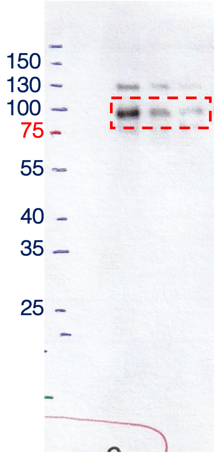

Supplementary Figure 6C OS9

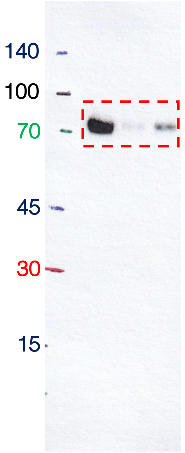

Supplementary Figure 6C HSP70
